# SARS-CoV-2 spike-specific T_FH_ cells exhibit unique responses in infected and vaccinated individuals

**DOI:** 10.1038/s41392-023-01650-x

**Published:** 2023-10-06

**Authors:** Rongzhang He, Xingyu Zheng, Jian Zhang, Bo Liu, Qijie Wang, Qian Wu, Ziyan Liu, Fangfang Chang, Yabin Hu, Ting Xie, Yongchen Liu, Jun Chen, Jing Yang, Shishan Teng, Rui Lu, Dong Pan, You Wang, Liting Peng, Weijin Huang, Velislava Terzieva, Wenpei Liu, Youchun Wang, Yi-Ping Li, Xiaowang Qu

**Affiliations:** 1 College of Basic Medical Sciences, Hengyang Medical School, University of South China & MOE Key Lab of Rare Pediatric Diseases, 421001 Hengyang, China; 2 Translational Medicine Institute, The First People’s Hospital of Chenzhou, Hengyang Medical School, University of South China, 423000 Chenzhou, China; 3 The Central Hospital of Shaoyang, 422000 Shaoyang, China; 4 Institute of Human Virology, Zhongshan School of Medicine, and Key Laboratory of Tropical Disease Control of Ministry of Education, Sun Yat-sen University, 501180 Guangzhou, China; 5 School of Public Health, University of South China, 421001 Hengyang, China; 6 National Institutes for Food and Drug Control, Key Laboratory of the Ministry of Health for Research on Quality and Standardization of Biotech Products, Key Laboratory of Biological Product Quality Research and Evaluation of National Medical Products Administration, 102629 Beijing, China; 7 Laboratory of OMICs Technologies, Institute of Biology and Immunology of Reproduction “Acad. Kiril Bratanov”, Bulgarian Academy of Sciences, Sofia, 1113 Bulgaria

**Keywords:** Vaccines, Infectious diseases

## Abstract

Long-term humoral immunity to SARS-CoV-2 is essential for preventing reinfection. The production of neutralizing antibody (nAb) and B cell differentiation are tightly regulated by T follicular help (T_FH_) cells. However, the longevity and functional role of T_FH_ cell subsets in COVID-19 convalescents and vaccine recipients remain poorly defined. Here, we show that SARS-CoV-2 infection and inactivated vaccine elicited both spike-specific CXCR3^+^ T_FH_ cell and CXCR3^−^ T_FH_ cell responses, which showed distinct response patterns. Spike-specific CXCR3^+^ T_FH_ cells exhibit a dominant and more durable response than CXCR3^−^ T_FH_ cells that positively correlated with antibody responses. A third booster dose preferentially expands the spike-specific CXCR3^+^ T_FH_ cell subset induced by two doses of inactivated vaccine, contributing to antibody maturation and potency. Functionally, spike-specific CXCR3^+^ T_FH_ cells have a greater ability to induce spike-specific antibody secreting cells (ASCs) differentiation compared to spike-specific CXCR3^−^ T_FH_ cells. In conclusion, the persistent and functional role of spike-specific CXCR3^+^ T_FH_ cells following SARS-CoV-2 infection and vaccination may play an important role in antibody maintenance and recall response, thereby conferring long-term protection. The findings from this study will inform the development of SARS-CoV-2 vaccines aiming to induce long-term protective immune memory.

## Introduction

Severe acute respiratory syndrome coronavirus 2 (SARS-CoV-2) is the causative agent of coronavirus disease 2019 (COVID-19), which poses a serious health threat and has had considerable socioeconomic consequences worldwide.^1,2^ Effective strategies are urgently needed to establish persistent and immune memory of the appropriate magnitude at the individual and population levels to prevent the continued spread of infection. Thus, understanding how immune memory is successfully established in vaccinated individuals and those who have recovered from COVID-19 is essential for rational vaccine design to elicit long-lasting humoral and cellular immune responses against SARS-CoV-2 and variants of concern (VOCs).

Both SARS-CoV-2 natural infection and vaccination have been reported to elicit robust humoral and cellular immune responses.^3–12^ The early appearance of neutralizing antibodies (nAbs) associated with less severe disease in acute COVID-19 and the persistence of nAbs in recovered individuals contribute to preventing reinfection by blocking virus entry.^13,14^ Endemic human coronaviruses (HCoV-229E, HCoV-OC43, HCoV-NL63, and HCoV-HKU1), which usually only infect the upper respiratory tract, frequently cause homologous reinfection, and this may be due to the short lifespan of the immunoglobins that they induce.^15^ Unlike endemic coronaviruses, the three highly pathogenic coronaviruses (SARS-CoV-1, MERS-CoV, and SARS-CoV-2) usually induce severe lower respiratory tract infection, consequently triggering full host immune defenses, and may elicit more persistent antibody responses in patients who recover from infection.^16–19^ These persistent and appropriate nAb levels play an important role in preventing reinfection with SARS-CoV-2 or emerging VOCs,^20^ although cases of reinfection with VOCs have been reported.^21–23^

The production and maturation of high-affinity antibodies and memory B cells, as well as long-lived plasma cell differentiation, mainly rely on germinal center (GC) reactions in secondary lymphoid tissues, which are tightly regulated by T follicular helper (T_FH_) cells.^24^ T_FH_ cells are specialized B helper cells that enable the proliferation, survival, and differentiation of GC B cells through the delivery of costimulatory molecules and cytokine signals.^25–28^ Circulating T_FH_ cells may serve as GC T_FH_ cell counterparts, as they express low levels of PD-1, ICOS, and Bcl6 and exhibit a memory phenotype.^29^ These characteristics of T_FH_ cells have been correlated with high-affinity antibody responses during virus infection and vaccination.^30–34^

In the acute phase of SARS-CoV-2 infection, large amounts of low-affinity antibodies are produced rapidly, and in parallel, antigen-specific CD4^+^ T cells and circulating T_FH_ cells appear, which in turn contribute to antibody production to combat infection.^4,13,35,36^ In COVID-19 convalescents, we and others have previously demonstrated that CXCR3^+^ T_FH_ cells directly correlate with anti-spike antibody responses,^37–39^ and that some epitope (HLA-DRB1*15:01/S751)-specific T_FH_ cells with a long half-life (T_1/2_ = 227 days) also predominantly exhibit a CXCR3^+^ CCR6^−^ phenotype.^40^ Several studies have also shown that CCR6^+^ T_FH_ cells (most of which are CXCR3^−^) predominate and are maintained longer after recovery in the observation period.^6,37,41,42^ The administration of the mRNA vaccine elicited high nAb levels and circulating T_FH_ cell responses similar to those seen in COVID-19 convalescents.^11,43^ More recently, several studies have shown that SARS-CoV-2 infection and mRNA vaccination efficiently induce robust GC reactions, GC-resident T_FH_ and B-cell responses, and long-lived plasma cells in bone marrow.^12,44–47^ These responses ensure antibody production to prevent reinfection or maintain the levels of circulating antibodies. Although severely impaired GC reactions were found in critically ill or dying patients, the majority of infected or vaccinated individuals were reported to have GC reactions in lymphoid tissues for several months, which may support the maturation and maintenance of high-affinity antibodies.^44,47–50^ Thus, addressing the longevity of antibody and T_FH_ cell responses, as well as the functional role of T_FH_ cells, in supporting the antibody response in COVID-19 convalescents and vaccinated subjects is urgently required to guide the development of long-term protective vaccines.

In this study, we performed a longitudinal investigation of the kinetics and longevity of spike-specific antibody and T_FH_ cell subset responses in SARS-CoV-2 infection and vaccination and addressed the functional roles in supporting memory B cell differentiation into antibody-secreting cell (ASCs), as well as antibody production. Our findings provide new insights into the development of interventions and vaccines against SARS-CoV-2 and VOCs.

## Result

### Persistence of spike-specific circulating T_FH_ cell and subset responses in COVID-19 convalescents

**Fig. 1:**
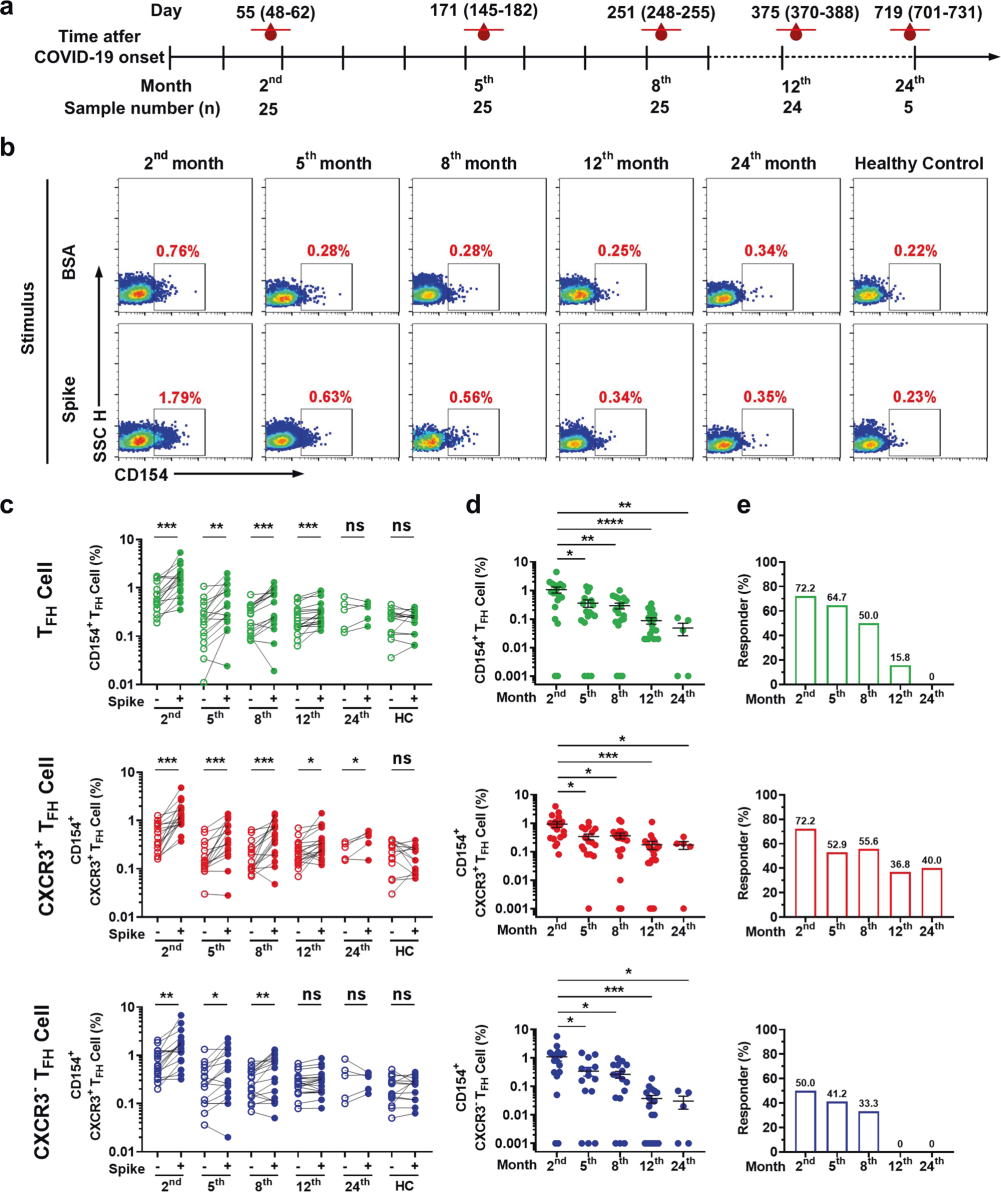
Longitudinal analysis of T_FH_ cell and subset responses in COVID-19 convalescents. **a** Timeline of blood sample collection from COVID-19 convalescents. The median and interquartile range (IQR) of days of sampling and number of samples are shown for each time point. **b** Representative flow plots of spike-specific T_FH_ cells (CD154^+^) upon BSA or spike protein stimulation. **c** Spike-specific T_FH_ cell, CXCR3^+^ T_FH_ cell, and CXCR3^−^ T_FH_ cell responses in COVID-19 convalescents at 2 months (*n* = 18), 5 months (*n* = 17), 8 months (*n* = 18), 12 months (*n* = 19), and 24 months (*n* = 5) after illness onset as well as in healthy controls (*n* = 14), upon stimulation. Paired *t* test was used to analyze the differences between the responses to BSA and spike protein stimulation. **d** Kinetics of spike-specific T_FH_ cell, CXCR3^+^ T_FH_ cell, and CXCR3^−^ T_FH_ cell responses (data presented with background subtracted; responses below background are shown as 0.001%) in COVID-19 convalescents at 2 months (*n* = 18), 5 months (*n* = 17), 8 months (*n* = 18), 12 months (*n* = 19), and 24 months (*n* = 5) after illness onset. Data are presented as the mean ± SEM. Mann–Whitney *U* test was used to analyze differences between the indicated time points. For (**c**, **d**), *P < 0.05; **P < 0.01; ***P < 0.001; ****P < 0.0001. *P* < 0.05 was considered to be a two-tailed significant difference, ns, not significant. **e** Percentage of spike-specific T_FH_ cell, CXCR3^+^ T_FH_ cell, and CXCR3^−^ T_FH_ cell responders (stimulation index > 2 was considered to be a positive response) among COVID-19 convalescents at 2 months (*n* = 18), 5 months (*n* = 17), 8 months (*n* = 18), 12 months (*n* = 19), and 24 months (*n* = 5) after illness onset

To longitudinally assess circulating T_FH_ cell and antibody responses after recovery from COVID-19, 104 blood samples were collected from 37 convalescents 2, 5, 8, 12, and 24 months after COVID-19 symptom onset (Fig. 1a and Supplementary Table 1). PBMCs were isolated and cultured for 24 h with stimulation in the presence of SARS-CoV-2 spike protein or BSA (5 μg/mL). Negative control PBMCs were collected from 14 healthy individuals before the COVID-19 pandemic and stimulated in the same manner. Circulating T_FH_ (CXCR5^+^ CD4^+^ CD3^+^ T) cells were gated, and antigen-specific T_FH_ cells were identified by CD154 (CD40L) assay (Supplementary Fig. 1). The results showed that the frequencies of CD154^+^ T_FH_ cells at 2, 5, 8, and 12 months, but not at 24 months, were significantly higher in the spike-stimulated group than in the BSA group; there was no difference in frequencies between the stimulation group and BSA group in healthy PBMCs (Fig. 1b, c, upper panel). Longitudinal analysis revealed that the spike-specific T_FH_ cell responses and the ratio of positive responders (stimulation index >2) declined gradually from months 2 to 24 (Fig. 1d, e, up panel). To explore the kinetics and longevity of T_FH_ cell subset responses, stimulated PBMCs were gated into CXCR3^+^ and CXCR3^−^ T_FH_ cell subsets, followed by analysis of CD154 expression (Supplementary Fig. 1). The results showed that the number of CD154^+^ CXCR3^+^ T_FH_ cells from months 2 to 24 was significantly enhanced after spike stimulation compared with BSA stimulation, while spike-responsive CXCR3^−^ T_FH_ cells were only seen from 2 to 8 months, and no significant changes were observed in CD154^+^ CXCR3^−^ T_FH_ cell frequency from 12 to 24 months (Fig. 1c, middle and bottom panels). Because most of the COVID-19 patients included in our study had been vaccinated by 24 months, only five COVID-19 convalescents who remained unvaccinated were available for analysis at this time point. Therefore, we confirmed the finding at 24 months using an alternative assay (Supplementary Fig. 2). Longitudinally, the spike-specific CXCR3^+^ T_FH_ cell responses and the ratio of positive responders declined from the 2nd to the 5th month but remained relatively steady from the 5th to the 24th month, while the number of spike-specific CXCR3^−^ T_FH_ cells, similar to spike-specific T_FH_ cells, declined slowly from months 2 to 24 (Fig. 1d, e, middle and bottom panel). Together, these findings demonstrate that spike-specific CXCR3^+^ and CXCR3^−^ T_FH_ cells exhibit distinct kinetics, and that spike-specific CXCR3^+^ T_FH_ cell responses may persist for more than 2 years in COVID-19 convalescents.

### Dynamics of spike-specific antibody responses in COVID-19 convalescents and correlations with T_FH_ cell frequency

**Fig. 2:**
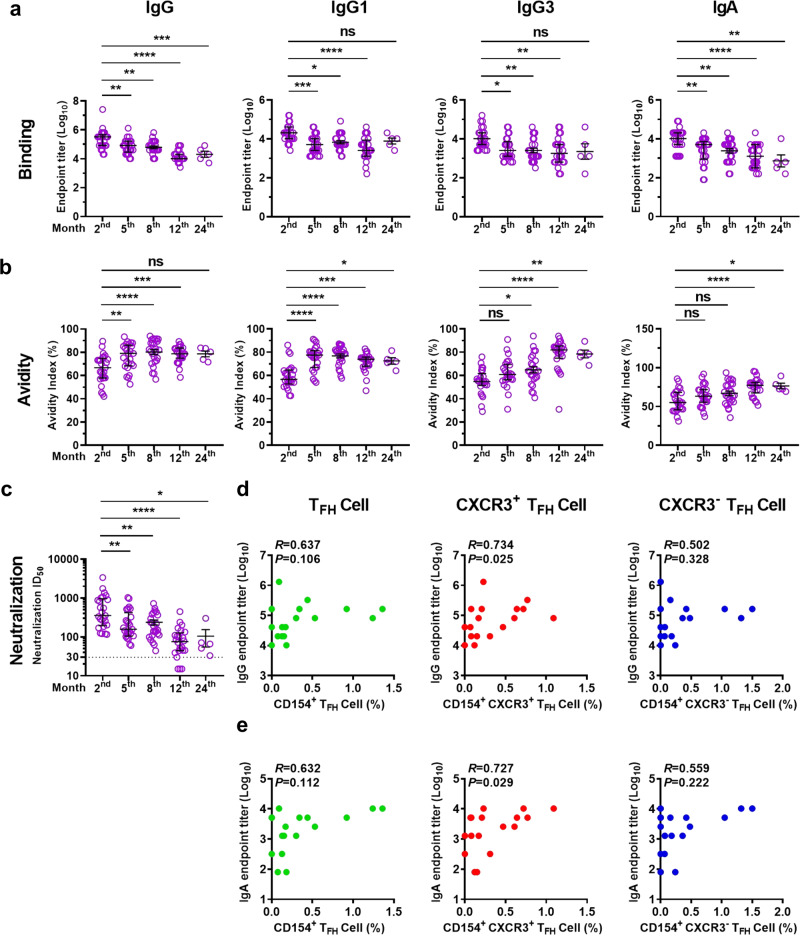
Kinetics of spike-specific antibody responses in COVID-19 convalescents. **a** Endpoint titers and (**b**) avidity index of plasma spike-specific IgG, IgG1, IgG3, and IgA antibodies among COVID-19 convalescents at 2 months (*n* = 25), 5 months (*n* = 25), 8 months (*n* = 25), 12 months (*n* = 24), and 24 months (*n* = 5) after illness onset. The endpoint titer data were logarithmically transformed. **c** Neutralization titers of COVID-19 convalescent plasma against SARS-CoV-2 pseudotyped virus at the indicated time points. The cut-off value was defined as ID_50_ = 30, and samples with an ID_50_ > 30 were considered to have a neutralizing effect. For (**a**–**c**), data are presented as median ± IQR (25–75%). Mann–Whitney *U* test was used to analyze the differences between the indicated time points, *P < 0.05; **P < 0.01; ***P < 0.001; ****P < 0.0001. *P* < 0.05 was considered to be a two-tailed significant difference, ns, not significant. **d**–**e** Correlations of frequencies of spike-specific T_FH_ cells, CXCR3^+^ T_FH_ cells, and CXCR3^−^ T_FH_ cells (data presented with background subtracted) with IgG and IgA endpoint titers at 5 months after illness onset (*n* = 17). Spearman’s rank correlation coefficient was used to describe the association between the frequency of T_FH_ cells and subsets with the IgG or IgA endpoint titers. *P* < 0.05 was considered to be a two-tailed significant difference

To assess the kinetics of antibody responses in COVID-19 convalescents, we examined spike-specific antibodies in the plasma at 2, 5, 8, 12, and 24 months. The endpoint titers and avidity index of the spike-specific antibodies (immunoglobin A (IgA), IgG, and IgG subclasses (IgG1, IgG2, and IgG3)) were determined. Endpoint titers of spike-specific IgG, IgG1, IgG3, and IgA were detectable at each time point, but all declined significantly from 2 months to 5 months and then remained stable from 5 months to 24 months (Fig. 2a). Of note, spike-specific IgG2 was present at low or undetectable levels at each time point (data not shown). IgA antibody levels have been reported to decline rapidly, and most are short-lived.^51^ Here, IgA antibodies were found to persist for at least 24 months in symptomatic convalescents (Fig. 2a). In contrast, the avidity of the spike-specific IgG, IgG1, IgG3, and IgA antibodies increased over time with different kinetics from endpoint titers, indicating that the antibodies continued to mature during the convalescent phase (Fig. 2b).

Next, we examined the neutralization activity of a plasma antibody against SARS-CoV-2 spike pseudotyped virus. The neutralization titers decreased significantly from 2 months to 5 months and then gradually declined from 5 months to 24 months (Fig. 2c). Within the first 8 months, all individuals showed nAb titers above the cut-off value (≥30); however, the titers then declined, with 87.5% (21 out of 24) and 100% (5 out of 5) maintained until 12 and 24 months, respectively (Fig. 2c). These data revealed that the nAb level dropped in the early phase of recovery, while maintained at lower levels for a long term, which is consistent with the dynamics of the antibody endpoint titer (Fig. 2a). The neutralization titers were positively associated with the endpoint titers of IgG, IgG1, IgG3, and IgA from 2 months to 12 months; less significant correlations were found at 24 months, because this time point only included five samples (Supplementary Fig. 3). These results suggest that spike-specific IgG and IgA responses both persisted for at least 2 years in the vast majority of convalescents and contributed to the neutralization effect.

The frequencies of spike-specific T_FH_ cells and their subsets, as well as their antibody titers, declined gradually in a similar pattern over the observation period (Figs. 1, 2). Thus, we further analyzed the correlations among spike-specific T_FH_ cells or their subsets and antibody titers at each time point. The endpoint titers for IgG and IgA were positively correlated with the proportions of CD154^+^ CXCR3^+^ T_FH_ cells but not CD154^+^ T_FH_ cells and CD154^+^ CXCR3^−^ T_FH_ cells at 5 months, while this correlation was not found at other time points (Fig. 2d, e and Supplementary Fig. 4), which indicated the well balance between T_FH_ cell response and antibody response only observed at 5 months. Together, these results further demonstrate the potential role of spike-specific T_FH_ cells in supporting high-affinity antibody maintenance in COVID-19 convalescents.

### Longitudinal analysis of spike-specific T_FH_ cell and antibody responses in inactivated vaccine recipients

**Fig. 3:**
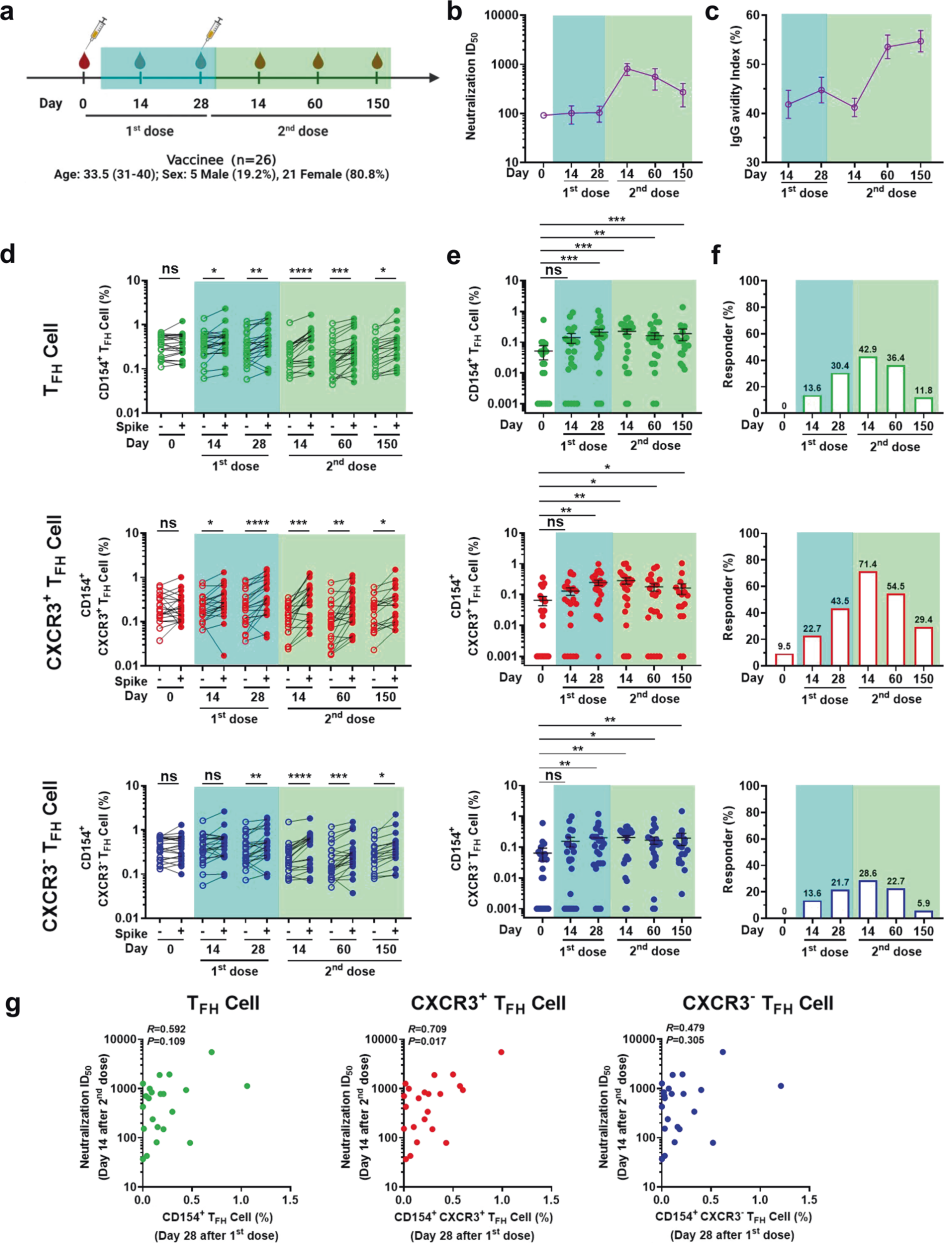
Kinetics of spike-specific antibody and circulating T_FH_ cell responses in inactivated vaccine recipients. **a** Timeline of vaccination and blood sampling. **b** nAb titers against pseudotyped SARS-CoV-2 spike virus at the indicated time points (day 0, *n* = 26; day 14 after 1st dose, *n* = 26; day 28 after 1st dose, *n* = 26; day 14 after 2nd dose, *n* = 26; day 60 after 2nd dose, *n* = 26; day 150 after 2nd dose, *n* = 26). Data are presented as the mean ± SEM. **c** The kinetics of spike-specific IgG avidity at the indicated time points (day 14 after 1st dose, *n* = 26; day 28 after 1st dose, *n* = 26; day 14 after 2nd dose, *n* = 26; day 60 after 2nd dose, *n* = 25; day 150 after 2nd dose, *n* = 25). Data are presented as the mean ± SEM. **d** Frequency of spike-specific T_FH_ cells, CXCR3^+^ T_FH_ cells, and CXCR3^−^ T_FH_ cells before vaccination (day 0, *n* = 21) and after vaccination (day 14 after 1st dose, *n* = 22; day 28 after 1st dose, *n* = 23; day 14 after 2nd dose, *n* = 21; day 60 after 2nd dose, *n* = 22; day 150 after 2nd dose, *n* = 17), upon stimulation. Paired *t* test was used to analyze the differences in T_FH_ cell and subset responses between BSA or spike protein stimulation at the indicated time points. **e** Kinetics of spike-specific T_FH_ cell, CXCR3^+^ T_FH_ cell, and CXCR3^−^ T_FH_ cell responses (data presented with background subtracted; responses below background are shown as 0.001%) before vaccination (day 0, *n* = 21) and after vaccination (day 14 after 1st dose, *n* = 22; day 28 after 1st dose, *n* = 23; day 14 after 2nd dose, *n* = 21; day 60 after 2nd dose, *n* = 22; day 150 after 2nd dose, *n* = 17). Data are presented as the mean ± SEM, Mann–Whitney *U* test was used to analyze the differences between the indicated time points. For (**d**–**e**), *P < 0.05; **P < 0.01; ***P < 0.001; ****P < 0.0001. *P* < 0.05 was considered to be a two-tailed significant difference, ns, not significant. **f** Percentage of spike-specific T_FH_ cell, CXCR3^+^ T_FH_ cell, and CXCR3^−^ T_FH_ cell responders (stimulation index > 2 was considered to be a positive response) before vaccination (day 0, *n* = 21) and after vaccination (day 14 after 1st dose, *n* = 22; day 28 after 1st dose, *n* = 23; day 14 after 2nd dose, *n* = 21; day 60 after 2nd dose, *n* = 22; day 150 after 2nd dose, *n* = 17). **g** Correlations among frequencies of spike-specific T_FH_ cells, CXCR3^+^ T_FH_ cells, and CXCR3^−^ T_FH_ cells (data presented with background subtracted) on day 28 after 1st dose and nAb titers on day 14 after 2nd dose (*n* = 22). Spearman’s rank correlation coefficient was used to describe the association between the frequency of T_FH_ cells and subsets with the nAb titer. *P* < 0.05 was considered to be a two-tailed significant difference

As described above, SARS-CoV-2 infection elicited persistent and highly correlated antibody and T_FH_ cell responses, and these responses were maintained for up to 2 years. To assess the dynamics of nAb and T_FH_ cell responses following vaccination, we recruited 26 participants who had completed the standard vaccination procedure involving two doses of inactivated vaccine (Sinovac) and collected blood samples at multiple time points to analyze the antibody titers and T_FH_ cell responses (Fig. 3a and Supplementary Table 2). The nAb titer peaked 14 days after the second vaccination dose, and then rapidly decreased until 150 days after the second vaccination dose (Fig. 3b). Unlike the neutralization kinetics, the IgG avidity was low at day 14 and 28 after the first dose and day 14 after second dose and then plateaued from day 60 through day 150 after the second dose (Fig. 3c). Notably, the peak of the vaccine-derived IgG antibody avidity index was lower than that of natural infection with SARS-CoV-2 at 8 months (natural infection peak index versus vaccination index: median, IQR: 82.00%, 72.42–90.21% vs. 55.37%, 49.93–61.15%). These findings suggest that two-dose immunization with inactivated vaccine elicited production of a lower level of nAbs that were not fully mature (low avidity) compared with natural infection with SARS-CoV-2.

Next, we assessed spike-specific T_FH_ cell and subset responses to vaccination. The frequencies of spike-specific T_FH_ cells and CXCR3^+^ T_FH_ cells significantly increased upon spike protein stimulation at day 14, while the number of spike-specific CXCR3^−^ T_FH_ cells increased at day 28 after the first dose, and the responses lasted throughout the observation period (Fig. 3d, left panel). Longitudinally, the responses of spike-specific T_FH_ cells, CXCR3^+^ T_FH_ cells, and CXCR3^−^ T_FH_ cells increased compared with day 0 and peaked at 14 days after the second vaccination dose (Fig. 3e). Interestingly, the ratio of spike-specific CXCR3^+^ T_FH_ cell positive responders increased dramatically and peaked at 14 days after the second vaccination dose (Fig. 3f). To explore the potential link between T_FH_ cells and antibody responses in inactivated vaccine recipients, we analyzed the relationships between spike-specific T_FH_ cells, CXCR3^+^ T_FH_ cells, and CXCR3^−^ T_FH_ cells and nAbs at each time point. We found frequencies of spike-specific CXCR3^+^ T_FH_ cells before the second dose (28 days after the first dose) were positively correlated with the peak nAb titers 14 days after the second dose, while no such correlations between nAb titers and spike-specific T_FH_ cells or CXCR3^−^ T_FH_ cells were observed (Fig. 3g). This indicates that early primed T_FH_ cells, especially spike-specific CXCR3^+^ T_FH_ cells after first dose, contribute to antibody production in the 14 days after the second dose, highlighting a key role for spike-specific CXCR3^+^ T_FH_ cells in the early activation of CD4^+^ T cells. These results suggest that two doses of inactivated vaccine can efficiently elicit nAb production and activate T_FH_ cell responses.

### A third booster dose augments the T_FH_ cell response and promotes spike-specific antibody potency and affinity maturation

**Fig. 4:**
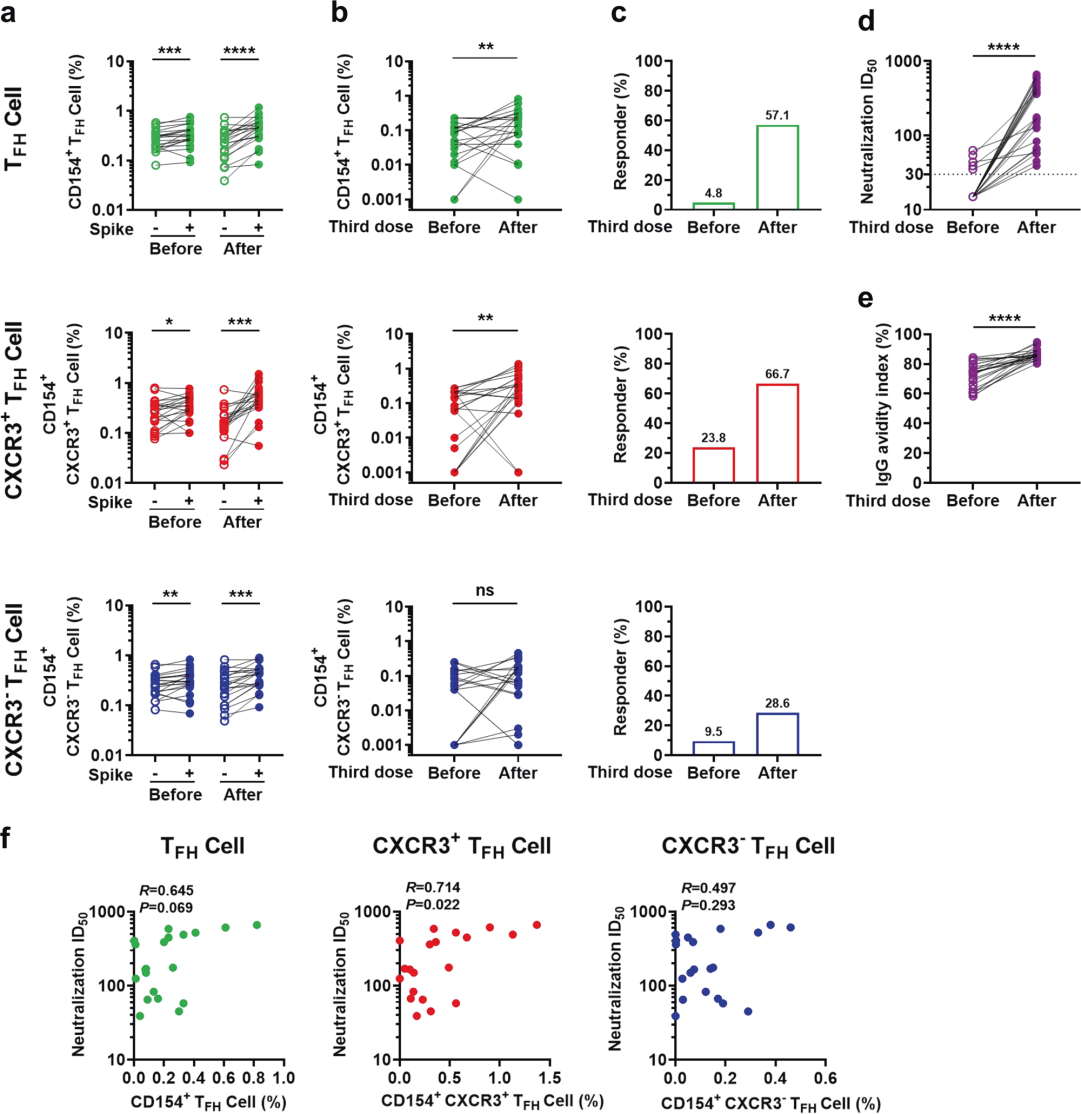
A third vaccine dose promoted spike-specific antibody potency and maturation. **a** Frequencies of spike-specific T_FH_ cells, CXCR3^+^ T_FH_ cells, and CXCR3^−^ T_FH_ cells before and after the third booster dose upon BSA or spike protein stimulation (*n* = 21). **b** Comparison of frequencies of spike-specific T_FH_ cells, CXCR3^+^ T_FH_ cells, and CXCR3^−^ T_FH_ cells (data presented with background subtracted; responses below background are shown as 0.001%) before and after the third booster dose (*n* = 21), upon spike protein stimulation. **c** Percentage of spike-specific T_FH_ cell, CXCR3^+^ T_FH_ cell, and CXCR3^−^ T_FH_ cell responders (stimulation index > 2 was considered to be a positive response) before and after the third booster dose (*n* = 21). **d** Neutralization titers before and after the third booster dose (*n* = 24). The cut-off value was defined as ID_50_ = 30, and ID_50_ > 30 was considered to indicate a neutralizing effect. **e** Spike-specific IgG avidity index before and after the third booster dose (*n* = 24). For (**a**, **b**), and (**d**, **e**), paired *t* test was used to analyze the differences between the two groups, *P < 0.05; **P < 0.01; ***P < 0.001; ****P < 0.0001. *P* < 0.05 was considered to be a two-tailed significant difference, ns, not significant. **f** Correlation of the frequencies of spike-specific T_FH_ cells, CXCR3^+^ T_FH_ cells, and CXCR3^−^ T_FH_ cells (data presented with background subtracted) with neutralization titers after the third booster dose (*n* = 21). Spearman’s rank correlation coefficient was used to describe the association between the frequency of T_FH_ cells and subsets and the nAb titer. *P* < 0.05 was considered to be a two-tailed significant difference

Because the nAb produced in response to the standard two-dose vaccination regimen waned significantly after 6 months, a third booster dose was recommended. Previous studies have shown that COVID-19 vaccine boosters elevate antibody responses, but it remains unclear how the T_FH_ cell response helps enhance the antibody response.^52,53^ To test the antibody and T_FH_ cell responses before and after the third dose, we recruited 24 individuals who had received two vaccination doses at least 6 months later and collected PBMCs before and 14 days after the third booster dose (Sinovac) (Supplementary Table 2). Spike-specific T_FH_ cells, CXCR3^+^ and CXCR3^−^ T_FH_ cell subsets all responded to spike stimulation before and after the third dose (Fig. 4a); however, only the responses and responder ratio of spike-specific T_FH_ cells and CXCR3^+^ T_FH_ cells, but not spike-specific CXCR3^−^ T_FH_ cells, expanded dramatically in response to the third dose (Fig. 4b, c). The third dose dramatically increased the neutralization titer by an average of more than 12-fold (Fig. 4d) and significantly promoted antibody affinity maturation (Fig. 4e), which reached a level similar to that induced by natural infection (natural infection peak index versus vaccination index: median, IQR: 82.00%, 72.42–90.21% vs. 86.11%, 84.99–88.42%). Furthermore, the frequencies of spike-specific CXCR3^+^ T_FH_ cells, but not spike-specific T_FH_ and CXCR3^−^ T_FH_ cells, after the third dose were significantly correlated with neutralization titers after the booster dose (Fig. 4f), indicating that the spike-specific T_FH_ cells, especially spike-specific CXCR3^+^ T_FH_ cells, elicited by the two-dose vaccination regimen further supported antibody production following the third dose. Thus, these findings show that a third vaccine dose augmented the antibody response and induced expansion of spike-specific T_FH_ cells and their subsets, preferentially spike-specific CXCR3^+^ T_FH_ cells, which may contribute to the enhancement of antibody quality and quantity. Of note, although no significant expansion in spike-specific CXCR3^−^ T_FH_ cells was observed after the booster dose, these cells still remained at comparable levels with spike-specific CXCR3^+^ T_FH_ cells, suggesting that they may co-contribute to antibody elevation.

### Omicron subvariants show distinct escape patterns corresponding to vaccine-elicited specific T_FH_ cell responses

**Fig. 5:**
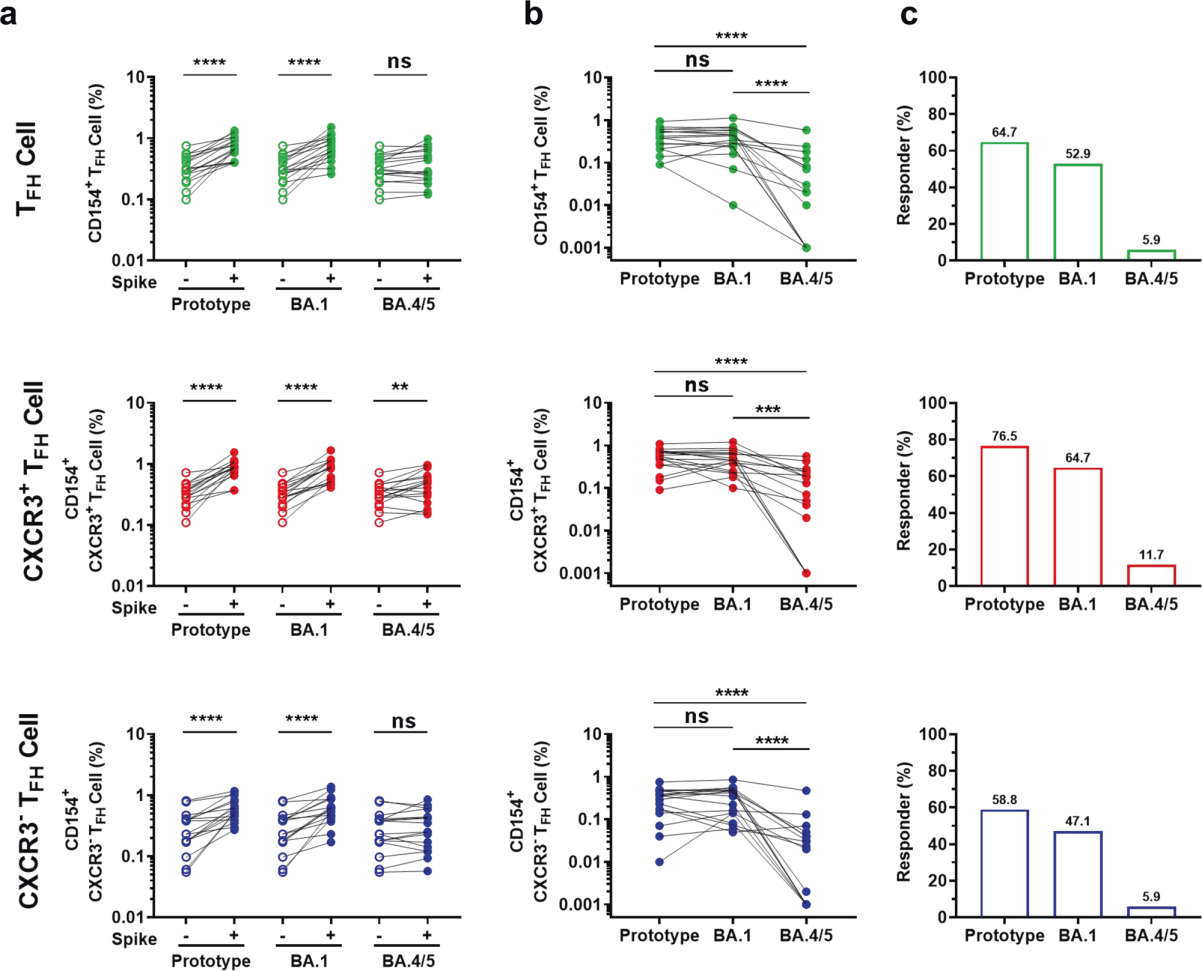
Vaccine-elicited T_FH_ cell responses to Omicron subvariants. **a** Frequencies of vaccine-elicited spike-specific T_FH_ cell, CXCR3^+^ T_FH_ cell, and CXCR3^−^ T_FH_ cell responses upon BSA, prototype, BA.1, or BA.4/5 spike protein stimulation (*n* = 17). **b** Comparison of spike-specific T_FH_ cell, CXCR3^+^ T_FH_ cell, and CXCR3^−^ T_FH_ cell responses (data presented with background subtracted; responses below background are shown as 0.001%) upon prototype, BA.1, or BA.4/5 spike protein stimulation (*n* = 17). Data are presented as the mean ± SEM. **c** Percentage of spike-specific T_FH_ cell, spike-specific CXCR3^+^ T_FH_ cell, and CXCR3^−^ T_FH_ cell responders (stimulation index > 2 was considered to be a positive response) upon prototype, BA.1, or BA.4/5 spike protein stimulation (*n* = 17). For (**a**, **b**), paired *t*-test was used to analyze the differences between the two groups, *P < 0.05; **P < 0.01; ***P < 0.001; ****P < 0.0001. *P* < 0.05 was considered to be a two-tailed significant difference, ns, not significant

Recent SARS-CoV-2 variants, especially the Omicron subvariants, have spread throughout the population, escaping vaccine-elicited antibody responses and consequently caused breakthrough infections.^22,23^ Whether these subvariants also escape vaccine-elicited T_FH_ cell responses is unclear. Here, we tested the vaccine-elicited T_FH_ cell response to two representative Omicron subvariants, BA.1 and BA.4/5. PBMCs from 17 vaccinees who had received two or three doses of inactivated vaccine (14 days after the second or third booster dose) were stimulated with BSA or with spike protein from a prototype SARS-CoV-2 (Wuhan-Hu-1) strain and Omicron subvariants BA.1, or BA.4/5 (Supplementary Table 3). Similar to prototype spike protein stimulation, BA.1 spike protein stimulation also expanded significant specific T_FH_ cell, CXCR3^+^ T_FH_ cell, and CXCR3^−^ T_FH_ cell responses compared with BSA stimulation; however, BA.4/5 spike protein stimulation only induced a significant specific CXCR3^+^ T_FH_ cell response (Fig. 5a). Vaccine-elicited spike-specific T_FH_ cells, CXCR3^+^ T_FH_ cells, and CXCR3^−^ T_FH_ cells showed a distinct recall response to prototype spike, BA.1, and BA.4/5 spike protein stimulation. Prototype and BA.1 spike protein stimulation elicited similar spike-specific T_FH_ cell and subset responses, while BA.4/5 spike protein stimulation induced significantly lower vaccine-elicited T_FH_ cell responses (Fig. 5b). Furthermore, the ratio of positive responders decreased slightly in response to BA.1 spike protein stimulation, but dropped dramatically in response to BA.4/5 spike protein stimulation, in comparison to prototype spike protein stimulation (Fig. 5c). These results suggest that vaccine-elicited T_FH_ cell memory exhibits distinct response patterns to Omicron BA.1 and BA.4/5, even though multiple mutations have accumulated in both variant spikes. This pattern is consistent with the observation that BA.1 partially escapes the vaccine-elicited antibody response, while BA.4/5 exhibits more serious escape, even though BA.1 infection elicited antibody responses.^21–23^ These findings suggest that the evolution of SARS-CoV-2 variants gradually enables them to escape the antibody and T_FH_ cell responses elicited by previous infection or vaccination.

### Spike-specific CXCR3^+^ T_FH_ and CXCR3^−^ T_FH_ cells exhibit distinct activation statuses and IL-21 secretion capacity

We have shown that spike-specific CXCR3^+^ T_FH_ cells and spike-specific CXCR3^−^ T_FH_ cells exhibit distinct responsiveness and persistence in both convalescents and vaccinees (Figs. 1, 3, 4). To further assess the functional differences between the spike-specific T_FH_ cell subsets, we compared the activation status of and secretion of IL-21 (a T_FH_ cell signature cytokine) by T_FH_ cells, especially CXCR3^+^ T_FH_ cells and CXCR3^−^ T_FH_ cells upon antigen exposure.

**Fig. 6:**
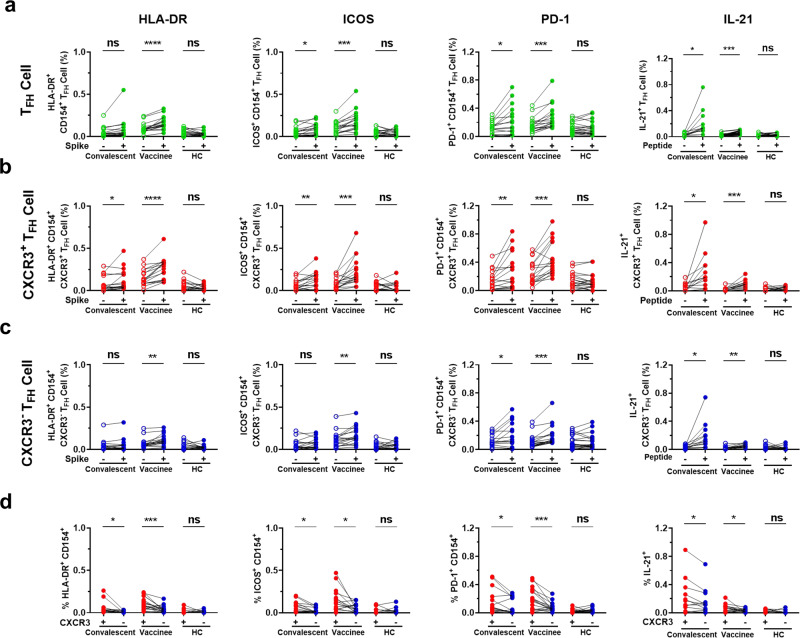
Spike-specific CXCR3^+^ T_FH_ cells exhibited higher activation status and greater IL-21 secretion than spike-specific CXCR3^−^ T_FH_ cells upon antigen stimulation. **a**–**c** Frequencies of HLA-DR^+^ CD154^+^, ICOS^+^ CD154^+^, PD-1^+^ CD154^+^ expression and intracellular IL-21 secretion by T_FH_ cells, CXCR3^+^ T_FH_ cells, and CXCR3^−^ T_FH_ cells from convalescents, vaccinees, and healthy controls upon control or antigen stimulation. **d** Comparison of HLA-DR^+^ CD154^+^, ICOS^+^ CD154^+^, PD-1^+^ CD154^+^, and intracellular IL-21 expression (data presented with background subtracted) among CXCR3^+^ and CXCR3^−^ T_FH_ cells from convalescents, vaccinees, and healthy controls, upon antigen stimulation. Frequencies of HLA-DR^+^ CD154^+^, ICOS^+^ CD154^+^, and PD-1^+^ CD154^+^ cells among T_FH_ cells, CXCR3^+^ T_FH_ cells, and CXCR3^−^ T_FH_ cells from convalescents (*n* = 16), vaccinees (*n* = 17), and healthy controls (*n* = 19) upon BSA or spike protein stimulation. Frequencies of intracellular IL-21 expression by T_FH_ cells, CXCR3^+^ T_FH_ cells, and CXCR3^−^ T_FH_ cells from convalescents (*n* = 12), vaccinees (*n* = 13), and healthy controls (*n* = 11) upon SARS-CoV-2 peptide pool stimulation. Paired *t*-test was used to analyze the differences between the two groups. *P < 0.05; **P < 0.01; ***P < 0.001; ****P < 0.0001. *P* < 0.05 was considered to be a two-tailed significant difference; ns, not significant

To investigate the activation status (HLA-DR, ICOS, and PD-1) of T_FH_ cells, PBMCs from 16 COVID-19 convalescents, 17 vaccinees, and 19 healthy controls were stimulated with BSA or spike protein (Supplementary Table 4). The results showed that the proportions of HLA-DR^+^ CD154^+^, ICOS^+^ CD154^+^, and PD-1^+^ CD154^+^ cells among T_FH_ cells, CXCR3^+^ T_FH_ cells, and CXCR3^−^ T_FH_ cells were significantly enhanced upon spike protein stimulation compared with BSA stimulation in both COVID-19 convalescents and vaccinees, except for the proportions of HLA-DR^+^ CD154^+^ in T_FH_ cells and CXCR3^−^ T_FH_ cells, as well as ICOS^+^ CD154^+^ cells in CXCR3^−^ T_FH_ cells, from convalescents (Fig. 6a–c). There were no changes in the activation status of T_FH_ cells or subsets in healthy controls upon spike protein stimulation (Fig. 6a–c). Furthermore, spike-specific CXCR3^+^ T_FH_ cells were more highly activated than spike-specific CXCR3^−^ T_FH_ cells in both COVID-19 convalescents and vaccinees upon spike protein stimulation, while there was no difference in healthy controls (Fig. 6d). These results suggest that SARS-CoV-2 infection and vaccination elicit an expansion of spike-specific T_FH_ cells that can be reactivated upon antigen exposure, and that spike-specific CXCR3^+^ T_FH_ cells are more highly activated than CXCR3^−^ T_FH_ cells upon antigen exposure.

IL-21 secreted by T_FH_ cells is critical in supporting B cell differentiation and antibody production.^25,54^ Therefore, to further explore the functional difference in spike-specific CXCR3^+^ and CXCR3^−^ T_FH_ cells, PBMCs from 12 COVID-19 convalescents, 13 vaccinees, and 11 healthy controls (Supplementary Table 4) were stimulated with spike peptide pools. Intracellular IL-21 secretion was significantly increased in T_FH_ cells, CXCR3^+^ T_FH_ cells, and CXCR3^−^ T_FH_ cells from both COVID-19 convalescents and vaccinees, but not from healthy controls, upon peptide stimulation (Fig. 6a–c, right panel). Additionally, we found that intracellular IL-21 secretion was prominent in convalescents, while it was relatively low in vaccinees (Fig. 6d, right panel). Intriguingly, the amount of IL-21 secreted by CXCR3^+^ T_FH_ cells was significantly higher than that secreted by CXCR3^−^ T_FH_ cells from both COVID-19 convalescents and vaccinees, but not from healthy controls, upon peptide stimulation (Fig. 6d, right panel). The higher activated status of and IL-21 secretion by CXCR3^+^ T_FH_ cells compared with CXCR3^−^ T_FH_ cells upon antigen exposure indicates that spike-specific CXCR3^+^ T_FH_ cells may play a greater role in supporting B cell function than spike-specific CXCR3^−^ T_FH_ cells in both COVID-19 convalescents and vaccinees.

### Spike-specific CXCR3^+^ T_FH_ cells show superior capacity to spike-specific CXCR3^−^ T_FH_ cells in supporting antibody-secreting cell differentiation in both COVID-19 convalescents and vaccinees

**Fig. 7:**
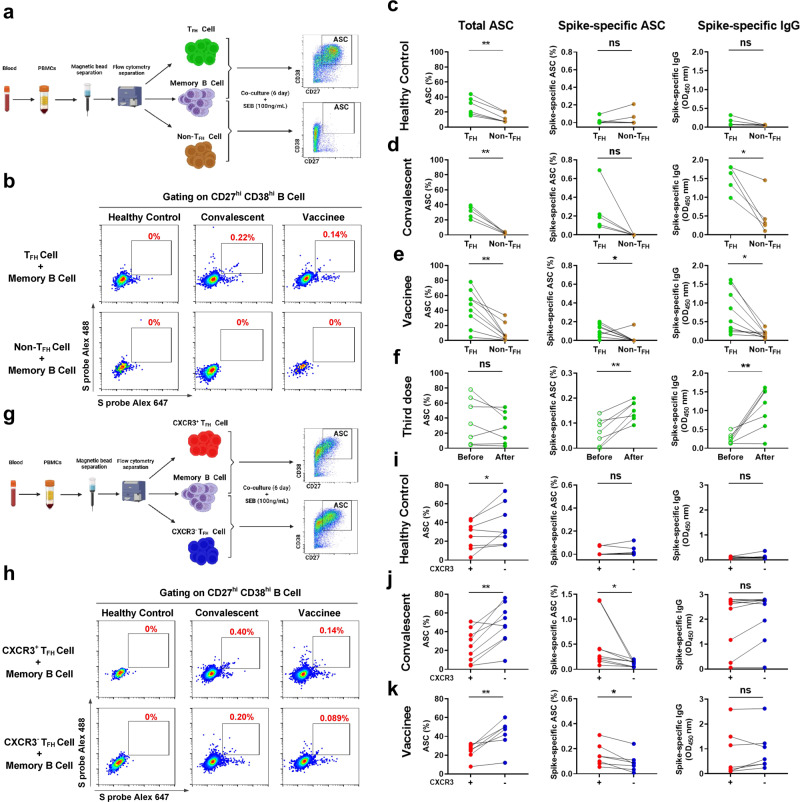
Spike-specific CXCR3^+^ T_FH_ cells were more able than spike-specific CXCR3^−^ T_FH_ cells to support ASCs differentiation in vitro. **a** Diagram of T_FH_ cells or non-T_FH_ cells coculturing with autologous memory B cells. **b** Representative flow plots of spike-specific ASCs after coculturing T_FH_ cells or non-T_FH_ cells with autologous memory B cells from healthy controls, convalescents, and vaccinees in the presence of SEB (100 ng/mL) for 6 days. **c**–**e** Comparison of total ASCs, spike-specific ASCs, and spike-specific IgG in the supernatant between T_FH_ and non-T_FH_ cells after coculture with autologous memory B cells from healthy controls (*n* = 6), convalescents (*n* = 5), and vaccinees (*n* = 9). **f** Comparison of the total ASCs, spike-specific ASCs, and spike-specific IgG from before (*n* = 7) and after (*n* = 7) the third booster dose. **g** Diagram of CXCR3^+^ T_FH_ cells or CXCR3^−^ T_FH_ cells coculturing with autologous memory B cells. **h** Representative flow plots of spike-specific ASCs after coculturing of CXCR3^+^ or CXCR3^−^ T_FH_ cells with autologous memory B cells from healthy controls, convalescents, and vaccinees in the presence of SEB (100 ng/mL) for 6 days. **i**–**k** Comparison of frequencies of total ASCs, spike-specific ASCs, and spike-specific IgG in the supernatant between CXCR3^+^ T_FH_ cells and CXCR3^−^ T_FH_ cells after coculturing with autologous memory B cells from healthy controls (*n* = 8), convalescents (*n* = 9), and vaccinees (*n* = 7) in the presence of SEB (100 ng/mL) for 6 days. For (**c**–**f**) and (**i**–**k**), paired *t* test was used to analyze the differences between the two groups. *P < 0.05; **P < 0.01. *P* < 0.05 was considered to be a two-tailed significant difference; ns, not significant

Previous studies have shown that both spike-specific CD4^+^ T cells and T_FH_ cells are associated with antibody production in SARS-CoV-2 infection.^5,37–39,55^ To discriminate between the functional roles that these cells play in supporting ASC differentiation and antibody production, we cocultured T_FH_ (CXCR5^+^ CD4^+^ CD3^+^ T) cells or non-T_FH_ (CXCR5^−^ CD4^+^ CD3^+^ T) cells from healthy controls (*n* = 6), convalescents (*n* = 5), and vaccine recipients (*n* = 9) with autologous memory B cells (5 × 10^4^ cells for each cell type) for 6 days in the presence of staphylococcal enterotoxin B (SEB) (Supplementary Table 5). Total ASCs (CD38^hi^ CD27^hi^ CD19^+^ B cells) and spike-specific ASCs were measured by FACS (Fig. 7a, b), and spike-specific IgG in the supernatant was measured by ELISA after coculturing (Fig. 7c–f, right panel). In healthy controls, T_FH_ cells but not non-T_FH_ cells efficiently supported autologous memory B cells differentiation into total ASCs, and, as expected, spike-specific ASCs and IgG were rarely observed (Fig. 7c). T_FH_ cells from COVID-19 convalescents and vaccinees efficiently supported autologous memory B cells differentiation into both total ASCs and spike-specific ASCs and produced spike-specific IgG (Fig. 7d, e). Receiving a third booster dose of the vaccine (*n* = 7) further enhanced the humoral immune responses, as more spike-specific ASCs and IgG were produced than before receiving the booster dose (Fig. 7f). Moreover, the frequencies of spike-specific ASCs correlated with spike-specific IgG OD_450_ (Supplementary Fig. 5). Taken together, these findings suggest that T_FH_ cells, but not non-T_FH_ cells, are the major player in supporting antibody recall response in COVID-19 convalescents and vaccinees upon antigen re-exposure.

As described above, we found that spike-specific CXCR3^+^ T_FH_ cells were associated with antibody response magnitude and were more responsive than spike-specific CXCR3^−^ T_FH_ cells upon antigen stimulation in both COVID-19 convalescents and vaccinees (Figs. 1–6). However, the roles and functional differences between spike-specific CXCR3^+^ T_FH_ cells and spike-specific CXCR3^−^ T_FH_ cells in supporting ASC differentiation and antibody production remained undefined. To address this question, bulk CXCR3^+^ and CXCR3^−^ T_FH_ cells were sorted from COVID-19 convalescents (*n* = 9), vaccinees (*n* = 7), and healthy controls (*n* = 8) and cocultured with autologous memory B cells (Fig. 7g, h and Supplementary Table 5), respectively. After coculture, B cell differentiation and spike-specific antibody production were assessed. The results showed that CXCR3^−^ T_FH_ cells were more efficient than CXCR3^+^ T_FH_ cells in supporting memory B cell differentiation into total ASCs in healthy controls, convalescents, and vaccinees (Fig. 7i-k, left panel), which is consistent with a previous report that circulating human CXCR3^−^ T_FH_ cells are more efficient than CXCR3^+^ T_FH_ cells in supporting B cell differentiation.^29^ However, CXCR3^+^ T_FH_ cells showed superior capacity to CXCR3^−^ T_FH_ cells in supporting spike-specific ASCs differentiation in both COVID-19 convalescents and vaccinees (Fig. 7j, k, middle panel). No spike-specific ASCs differentiation or spike-specific antibody production was observed when CXCR3^+/−^ T_FH_ cells were cocultured with autologous memory B cells from healthy controls (Fig. 7i). We did not observe higher spike-specific IgG production in CXCR3^+^ T_FH_ cells compared with CXCR3^−^ T_FH_ cells from COVID-19 convalescents and vaccinees cocultured with autologous memory B cells, as expected (Fig. 7j, k, right panel). This might be due to the lower ex vivo proportion of CD154^+^ CXCR3^+^ T_FH_ cells within bulk CXCR3^+^ T_FH_ cells compared with CD154^+^ CXCR3^−^ T_FH_ cells within bulk CXCR3^−^ T_FH_ cells (Supplementary Fig. 6), although spike-specific CXCR3^+^ T_FH_ cells exhibited a more activated status, and greater IL-21 secretion than spike-specific CXCR3^−^ T_FH_ cells in convalescents and vaccinees (Fig. 6). These findings further confirm that spike-specific T_FH_ cell subsets, especially the spike-specific CXCR3^+^ T_FH_ cell subset, play a functional role in supporting antibody maintenance and recall response in natural infection and vaccination. Targeting T_FH_ cells, especially CXCR3^+^ T_FH_ cells, may therefore be a novel approach to enhancing the long-term efficacy of SARS-CoV-2 vaccines.^56^

## Discussion

In this study, we systemically investigated the longitudinal dynamics of spike-specific T_FH_ cell and antibody responses in individuals up to 2 years after COVID-19 recovery and in individuals who received two or three doses of inactivated vaccine. We discovered that spike-specific CXCR3^+^ T_FH_ cells are persistent and express higher levels of T_FH_ functional molecules than spike-specific CXCR3^−^ T_FH_ cells in convalescents and inactivated vaccine recipients. The spike-specific memory B cells generated by natural infection and vaccination was efficiently reactivated by spike-specific CXCR3^+^ T_FH_ cells and CXCR3^−^ T_FH_ cells to differentiate into ASCs and produce spike-specific antibodies. These findings suggest that SARS-CoV-2-specific long-lasting humoral immunity can be generated and recalled, thus providing new insight into COVID-19 immunity and how to elicit long-term protection.

Previous studies have shown that SARS-CoV-2 infection induces robust spike-specific T_H_1 and T_FH_ cell responses in the acute phase that are maintained in the convalescent phase and persist for several months to a year.^41,42,57^ Here, in a longitudinal analysis of circulating T_FH_ cells over a 2-year period in COVID-19 convalescents, we found that spike-specific CXCR3^+^ T_FH_ cell response persisted for up to 24 months or more. In most symptomatic COVID-19 cases, robust GC reactions occur and are maintained for at least 6 months.^47,50^ GC T_FH_ cells promote B cell maturation and differentiation into high-affinity memory B cells and long-lived plasma cells. The long-term persistence of circulating T_FH_ cell responses in COVID-19 convalescents may be attributed to active GC reactions in lymph nodes after recovery.

In line with the T_FH_ cells dynamics observed in this study, spike-specific IgG, IgG1, IgG3, and IgA antibody endpoint titers, as well as neutralization titers, showed a sharp decline from month 2 to month 5 and then were maintained at the same level in the majority of convalescents for at least 24 months, which is longer than the 16-month persistence reported recently.^20^ The initial sharp decrease was most likely due to the short half-life of serum antibodies and ASCs generated in the acute phase.^41^ ASCs normally decay within a few weeks, and only a smaller population of long-lived plasma cells can live from several months to years.^58^ IgA antibody has been shown to play a dominant role in neutralization in early infection, although it is short-lived and declines rapidly after infection.^51^ Here, we found that systematic spike-specific IgA antibodies were maintained for 24 months, although at a lower level. This is much longer than a previous observation of detectable IgA 6 months after infection, and may contribute to long-term protection.^59^ Antibody maturation is a relatively slow process that is supported by T_FH_ cells in the GC.^24^ Our result show that spike-specific IgG antibody maturation peaked at 5 to 8 months after infection, while spike-specific IgG3 and IgA were fully mature at the 12 months. This result is in line with an earlier observation in recovered SARS patients that antibody avidity continued to mature from low avidity in the early phase to high avidity at late phases of disease recovery.^60^ The maturation of antibody avidity also reflects the persistence of GC reactions after SARS-CoV-2 resolution.^50^ These findings indicate that long-term T_FH_ cell responses are required in COVID-19 convalescents to gradually support high-quality antibody maturation and maintenance.

Inactivated vaccines have been widely used and proven to elicit short-term humoral immunity when administered in two doses, thereby conferring protection from SARS-CoV-2 and VOCs infection or severe disease.^61,62^ In this study, we also found that immunization with inactivated vaccine elicited humoral immunity in a similar manner to natural infection, but that the magnitudes of the antibody and T_FH_ cell responses were significantly smaller. Although nAb levels peaked at 14 days and mature antibodies were observed 2 months after the second dose, spike-specific T_FH_ cells were detected 14 days after the first dose, earlier than the appearance of nAbs. However, the nAb level significantly declined 6 months after immunization. Based on the memory immunity generated by two-dose vaccination, administering a third booster dose significantly magnified the nAb responses and increased antibody avidity and the frequency of responsive T_FH_ cells. These results are consistent with several recent studies of inactivated vaccine showing that a third dose increased the antibody-neutralizing effect for SARS-CoV-2 and some VOCs, proving the utility and necessity of the booster dose.^62,63^ However, the T_FH_ cell and antibody responses elicited by inactivated vaccines seem to last only a short period of time, unlike natural infections, which elicit a lasting and relatively stable immune memory response.^57^ In addition, we found that recent circulating Omicron subvariants exhibited distinct vaccine-elicited spike-specific T_FH_ cell memory escape patterns: BA.1 maintained responsiveness to vaccine-elicited T_FH_ cells, while BA.4/5 partially escaped these T_FH_ cell responses, consistent with the antibody escape patterns of Omicron subvariants.^21–23^ Thus, systematically and continuously investigating the efficacy of vaccine-elicited T cell and antibody responses to emerging or pre-emergent variants is critical to inform vaccine improvement.

The long-lived antibody response is mainly supported by T_FH_ cells in natural infection and vaccination.^47,64^ We found that SARS-CoV-2 infection and vaccination induced a persistent spike-specific T_FH_ cell responses, consistent with other studies, thus laying the foundation for long-term immunity.^61,62^ Notably, spike-specific T_FH_ cells, especially spike-specific CXCR3^+^ T_FH_ cells, positively correlated with antibody responses at 5 months in convalescents, and the quantity and quality of the antibodies were well balanced. Spike-specific CXCR3^+^ T_FH_ cells elicited by the first vaccine dose also positively correlated with peak nAb titers, and this relationship was further strengthened by a third booster dose. Functionally, T_FH_ cells, but non-T_FH_ cells, from COVID-19 convalescents and vaccinees could efficiently recall autologous memory B cells to differentiate into spike-specific ASCs and produce antibodies. Therefore, circulating spike-specific T_FH_ cells are a surrogate of bona fide GC T_FH_ cells that support spike-specific ASC differentiation and antibody production in SARS-CoV-2 natural infection and vaccination.^29^ Of note, our study did not exclude the possibility that non-T_FH_ cells support short-lived plasmablast differentiation and produce low-affinity antibodies in the very early acute phase to constrain infection rapidly, as large amounts of antibodies are produced by extrafollicular B cells in some severe cases.^65^

SARS-CoV-2 infection-induced circulating T_FH_ cells exhibited a clear phenotypic bias towards a CCR6^+^ CXCR3^−^ phenotype, and this subset comprises the majority of spike-specific T_FH_ cells in natural infection.^6,37,41,42^ While we and others have previously shown that the CXCR3^+^ T_FH_ cell subset is increased in convalescents and is positively correlated with the spike-specific antibody response.^37–39^ The association of T_H_1-like (CXCR3^+^) T_FH_ cells with antibody quantity and quality has also been characterized in influenza vaccine recipients and in other chronic viral infections.^30,31,33,34,66^ Furthermore, we previously demonstrated that the CXCR3^+^ and CXCR3^−^ T_FH_ subsets have distinct phenotypes and functions in HCV infection.^34^ However, the functional difference of spike-specific CXCR3^+^ T_FH_ and CXCR3^−^ T_FH_ cells in SARS-CoV-2 infection and vaccination is largely unknown. In this study, we demonstrated that spike-specific CXCR3^+^ T_FH_ cells are more persistent than spike-specific CXCR3^−^ T_FH_ cells in COVID-19 convalescents. And, spike-specific CXCR3^+^ T_FH_ cells exhibited higher activation status and greater IL-21 secretion than spike-specific CXCR3^−^ T_FH_ cells in both COVID-19 convalescents and vaccinees upon antigen exposure. Coculture of CXCR3^+^ T_FH_ or CXCR3^−^ T_FH_ cells with autologous memory B cells from COVID-19 convalescents and vaccinees showed that CXCR3^+^ T_FH_ cells were more able than CXCR3^−^ T_FH_ cells to support spike-specific memory B cell differentiation into ASCs. These findings confirm that both spike-specific CXCR3^+^ T_FH_ cells and CXCR3^−^ T_FH_ cells play an efficient, functional role in promoting the antibody response in natural infection and vaccination. However, the T_H_1-polarizing conditions of a viral infection or vaccination usually result in the predominant generation of T_H_1-like T_FH_ cells; this has been observed with influenza vaccination, live-attenuated yellow fever vaccination, HCV infection, and Zika virus infection.^30–32,34,67^ Given the importance of T_H_1-like T_FH_ cells in supporting the production of high-quality antibodies and the longevity of ASCs, strategies to promote T_H_1-like (CXCR3^+^) T_FH_ cell polarization would benefit SARS-CoV-2 vaccine development.

In conclusion, our study demonstrated that SARS-CoV-2 natural infection and vaccination elicit expansion of spike-specific CXCR3^+^ and CXCR3^−^ T_FH_ cell subsets, and that both subsets contribute to spike-specific high-affinity antibody maintenance and recall responses, but that they exhibited clear differences in persistence, functionality, and antibody-supporting ability. Spike-specific CXCR3^+^ T_FH_ cells are more persistent and superior to CXCR3^−^ T_FH_ cells in supporting antibody production, and may therefore confer long-term protection. These findings will inform vaccine design to provide long-term protection against SARS-CoV-2 and VOC infection by targeting T_FH_ cells, especially CXCR3^+^ T_FH_ cells.

### Limitations of the study

This study had some limitations: a single-marker (CD154) assay was used to identify antigen-specific T_FH_ cells, only five convalescent samples from the 24-month time point were available for analysis, and a limited number of vaccination cohort samples and Omicron subvariants were tested for immune escape capacity. The findings from the co-culture assay should be confirmed using spike protein or peptide pools.

## Materials and methods

### Study subjects

Two cohorts were included in this study: COVID-19 convalescents and vaccinated subjects. A total of 78 COVID-19 convalescents who had recovered from Wuhan-Hu-1 infection from January to March 2020 were recruited from The Central Hospital of Shaoyang, Hunan Province, China, and a subset of these individuals were followed for up to 24 months after symptom onset (Supplementary Tables 1, 4, and 5). In addition, 95 vaccinated subjects were recruited from The First People’s Hospital of Chenzhou, Hunan Province, China. All vaccinated subjects received at least two doses of inactivated vaccine (Sinovac, Beijing, China), while a subset of these subjects also received a third dose (Supplementary Tables 2–5). None of the enrolled COVID-19 convalescents had a history of SARS-CoV-2 vaccination, and none of the convalescents or vaccinees had subsequent SARS-CoV-2 or variant exposure or infection, as determined by frequent PCR testing and questionnaire administration during the observation period. For convalescent individuals, blood samples were collected 2, 5, 8, 12, and 24 months after COVID-19 symptom onset. For vaccine recipients, blood samples were collected before vaccination (day 0), 14 and 28 days after the first dose, and 14, 60, and 150 days after the second dose. For vaccinees who received a booster, blood samples were also taken before and 14 days after the third dose. Blood samples taken from healthy individuals before the COVID-19 pandemic were used as the negative control group. PBMCs and plasma were isolated and stored in liquid nitrogen and a −80 °C freezer, respectively. Each participant signed a written consent form. The study protocol was approved by the Institutional Ethical Review Board of The Central Hospital of Shaoyang (V.1.0, 20200301) and the First People’s Hospital of Chenzhou (V.3.0, 2021001), and the study was compliant with the “Guidance of the Ministry of Science and Technology (MOST), China”.

### Antibody endpoint titers

The endpoint titers of spike-specific antibodies were determined by measuring the binding activity of serially diluted plasma to the SARS-CoV-2 spike protein by ELISA. In brief, 96-well plates (Corning, NY, USA) were coated with SARS-CoV-2 spike protein (SARS-CoV-2 S1 + S2_ECD, 200 ng/well) (Sino Biological, Beijing, China) in PBS and incubated at 4 °C overnight. The plates were washed five times with PBS-T (0.05% Tween-20 in PBS) and then blocked with blocking buffer (2% FBS and 2% BSA in PBS-T) for 30 min. Two-fold serial dilutions of plasma, starting from a 1:20 dilution, were added to the 96-well plates in triplicate (100 µL/well) and incubated for 1 h at room temperature. Spike-specific antibodies were detected using horseradish peroxidase (HRP)-conjugated anti-human IgG (Jackson ImmunoResearch, PA, USA), IgG1, IgG3, and IgA (BaiaoTong Experiment Centre, Luoyang, China). Plasma samples collected from healthy subjects before the COVID-19 pandemic were used as the negative control group, and SARS-CoV-2 spike RBD-specific monoclonal antibody was generated in the laboratory and used as a positive control. Optical density at 450 nm (OD_450_) was measured for each reaction, and an OD_450_ value three-fold greater than the cut-off value (healthy control group) was considered a positive readout. The highest dilution showing a positive readout was defined as the endpoint titer of the antibody, and the data were logarithmically transformed.

### Antibody avidity assay

The avidity of spike-specific antibodies (IgG, IgG1, IgG3, and IgA) was measured using a modified two-step approach that we described previously.^39^ In the first step, plasma dilutions were optimized to obtain an OD_450_ value within the range of 0.5–1.5 to ensure linear measurement of antibody avidity. The second step was an ELISA that included elution with 1 M NaSCN. These measurements were performed in triplicate. The avidity index of an antibody was calculated as OD_NaSCN 1M_/OD_NaSCN 0M_ × 100%.

### Antibody neutralization assay

The neutralization activity of plasma was determined by reduction in luciferase expression after infecting Huh7 cells with pseudotyped virus, as described previously.^39^ In brief, SARS-CoV-2 pseudotyped virus was incubated in duplicate with serial dilutions of plasma samples (six dilutions: 1:30; 1:90; 1:270; 1:810; 1:2430; 1:7290) at 37 °C for 1 h. Then, freshly trypsinized cells were added, the mixture was incubated at 37 °C with 5% CO_2_ for 24 h, and the luminescence was measured. In parallel, control wells with virus only or cells only were included (six replicates). The background value in relative light units (RLUs) (wells with cells only) was subtracted from each value. Plasma from healthy controls was used as a negative control. Plasma from guinea pigs immunized with the SARS-CoV-2 spike protein was used as a positive control. The 50% inhibitory dilution (ID_50_) was defined as the plasma dilution that reduced the value in RLUs by 50% compared with the control wells (virus + cells). The cut-off value was defined as ID_50_ = 30, and samples with an ID_50_ > 30 were considered to have a neutralizing effect.

### Antigen-specific T_FH_ cell assay

To analyze spike-specific T_FH_ cells, a CD154 (CD40L) assay was used to assess the response of circulating T_FH_ cells to stimulation. In brief, cryopreserved PBMCs were thawed and allowed to recover in complete RPMI 1640 medium at 37 °C with 5% CO_2_ overnight. PBMCs (1 × 10^6^) were stimulated with SARS-CoV-2 spike protein (prototype: Wuhan-Hu-1) (S1 + S2_ECD, 5 μg/mL, Sino Biological, Beijing, China) or BSA (5 μg/mL, Sigma-Aldrich, St. Louis, MO, USA) at 37 °C with 5% CO_2_ for 24 h, and PE mouse anti-human CD154 (24-31) (BioLegend, San Diego, CA, USA) was added during the stimulation. To detect the vaccine-elicited T_FH_ cell and subset responses to Omicron subvariants, PBMCs from vaccinees were stimulated with BA.1 (5 μg/mL, Sino Biological, Beijing, China) or BA.4/5 (5 μg/mL, Sino Biological, Beijing, China) spike proteins. Concanavalin A (Con A, 5 μg/mL, Sigma-Aldrich, St. Louis, MO, USA) was used as a positive control. In parallel, PBMCs from healthy controls were stimulated under the same conditions. After stimulation, the cells were labeled with a LIVE/DEAD^®^ Fixable Blue Dead Cell Stain Kit (Thermo Fisher Scientific, Waltham, MA, USA) to identify dead cells and then treated with Fc Block (BioLegend, San Diego, CA, USA) to block nonspecific binding. The treated PBMCs were stained with antibodies that had been pretitrated to an optimized dilution and fluorescently labeled in 96-well V-bottom plates at 4 °C for 30 min. The fluorescently labeled antibodies used were as follows: BUV737 mouse anti-human CD4 (SK3), PerCP/Cyanine5.5 mouse anti-human CD3 (OKT3), APC mouse anti-human CXCR3 (G025H7) (BioLegend, San Diego, CA, USA), FITC mouse anti-human PD-1 (EH12.2H7) (BioLegend, San Diego, CA, USA), and PE-eFluor 610 mouse anti-human CXCR5 (MU5UBEE) (Thermo Fisher Scientific, Waltham, MA, USA). Samples were loaded onto a MoFlo XDP Flow Cytometer (Beckman Coulter, Brea, CA, USA) immediately after antibody staining. The cells were gated according to the following strategy (Supplementary Fig. 1): spike-specific T_FH_ cells (CD154^+^ CXCR5^+^ CD4^+^ CD3^+^ T cells), spike-specific CXCR3^+^ T_FH_ cells (CD154^+^ CXCR3^+^ CXCR5^+^ CD4^+^ CD3^+^ T cells), spike-specific CXCR3^−^ T_FH_ cells (CD154^+^ CXCR3^−^ CXCR5^+^ CD4^+^ CD3^+^ T cells). Gating was based on mean fluorescence intensity “minus one” (FMO) and an unstained control. All data were analyzed using FlowJo 10.0 software (Tree Star, San Carlos, CA, USA).

### T_FH_ cell activation detection

To determine the activation status of spike-specific T_FH_ cells, PBMCs were treated as described above. In brief, PBMCs (1 × 10^6^) were stimulated with SARS-CoV-2 spike protein (S1 + S2_ECD, 5 μg/mL, Sino Biological, Beijing, China) or BSA (5 μg/mL, Sigma-Aldrich, St. Louis, MO, USA) at 37 °C with 5% CO_2_ for 24 h, and PE mouse anti-human CD154 (24-31) (BioLegend, San Diego, CA, USA) was added during the stimulation. BUV737 mouse anti-human CD4 (SK3), PerCP/Cyanine5.5 mouse anti-human CD3 (OKT3), APC mouse anti-human CXCR3 (G025H7) (BioLegend, San Diego, CA, USA), FITC mouse anti-human PD-1 (EH12.2H7), APC/Cyanine7 mouse anti-human HLA-DR (L243) (BioLegend, San Diego, CA, USA), PE-eFluor 610 mouse anti-human CXCR5 (MU5UBEE), and PE-Cyanine7 mouse anti-human ICOS (ISA-3) (Thermo Fisher Scientific, Waltham, MA, USA) were used to stain the cells after stimulation. Gating was based on mean fluorescence intensity “minus one” (FMO) and unstained control. The following marker pairs were used to evaluate activation status of antigen-specific T_FH_ cell or subsets from COVID19 convalescents or vaccinees: HLA-DR^+^ CD154^+^, PD-1^+^ CD154^+^, and ICOS^+^ CD154^+^.

### Intracellular cytokine staining

PBMCs were stimulated for 6 h with SARS-CoV-2 peptide pools (2 μg/mL) (Mabtech, Stockholm, Sweden) or an equal amount of DMSO (3 μL/mL) in the presence of αCD28 (CD28.2, 1 μg/mL) and αCD49d (9F10, 1 μg/mL) costimulatory antibodies (Biolegend, San Diego, CA, USA). PMA (50 ng/mL) and ionomycin (1 μg/mL) stimulation was used as the positive control. Brefeldin A (5 µg/mL) was added after 1 h. After stimulation, the cells were labeled with a LIVE/DEAD® Fixable Blue Dead Cell Stain Kit (Thermo Fisher Scientific, Waltham, MA, USA) to identify dead cells and then treated with Fc Block (BioLegend, San Diego, CA, USA) to block nonspecific binding. Cells were surface-stained with BUV737 mouse anti-human CD4 (SK3) (BD Biosciences, Franklin Lake, NJ, USA), PerCP/Cyanine5.5 mouse anti-human CD3 (OKT3), APC mouse anti-human CXCR3 (G025H7) (BioLegend, San Diego, CA, USA), and PE-eFluor 610 mouse anti-human CXCR5 (MU5UBEE) (Thermo Fisher Scientific, Waltham, MA, USA) for 30 min in the dark at 4 °C. Then, the PBMCs were permeabilized using a Fixation/Permeablization Kit (BD biosciences, New Jersey, USA) according to the manufacturer’s instructions. The cells were then stained intracellularly with PE mouse anti-human IL-21 (3A3-N2) (BioLegend, San Diego, CA, USA). Gating was based on the mean fluorescence intensity “minus one” (FMO) and the unstained control. All data were analyzed using FlowJo 10.0 software (Tree Star, San Carlos, CA, USA).

### T_FH_ and memory B cell coculture

To test the functional role of T_FH_ cells, CXCR3^+^ and CXCR3^−^ T_FH_ cell subsets, and non-T_FH_ cells in supporting ASC differentiation and antibody production, these cells were co-cultured with autologous memory B cells. In detail, CD4^+^ T cells and CD19^+^ B cells were purified from PBMCs of COVID-19 convalescents and vaccinees using CD4 and CD19 MicroBeads (Miltenyi Biotec, Bergisch Gladbach, Germany), respectively. Purified CD4^+^ T cells and CD19^+^ B cells were stained with BUV737 mouse anti-human CD4 (SK3), PE mouse anti-human CXCR3 (1C6) (BD Biosciences, Franklin Lake, NJ, USA), PE-eFluor 610 mouse anti-human CXCR5 (MU5UBEE) (Thermo Fisher Scientific, Waltham, MA, USA), PE mouse anti-human CD20 (2H7), and PE-Cy7 mouse anti-human CD27 (M-T271) (BioLegend, San Diego, CA, USA) and then sorted by FACS as follows: T_FH_ cells (CXCR5^+^ CD4^+^ T cells), non-T_FH_ cells (CXCR5^−^ CD4^+^ T cells), CXCR3^+^ T_FH_ cells (CXCR3^+^ CXCR5^+^ CD4^+^ T cells), CXCR3^−^ T_FH_ cells (CXCR3^−^ CXCR5^+^ CD4^+^ T cells), and memory B cells (CD27^+^ CD20^+^ B cells). Sorted T_FH_, non-T_FH_, CXCR3^+^ T_FH_, and CXCR3^−^ T_FH_ cells (5 × 10^4^ cells for each type) were co-cultured with autologous memory B cells (5 × 10^4^ cells) in the presence of 100 ng/mL staphylococcal enterotoxin B (SEB) (Toxin Technology, Sarasota, FL, USA) and RPMI 1640 medium supplemented with 10% FBS in 96-well U-bottom plates for 6 days. After co-culture, spike-specific IgG in the supernatant was determined by ELISA. ASCs (CD27^hi^ CD38^hi^ CD4^−^ cells) and spike-specific ASCs (spike-probe FITC^+^ and spike-probe ALEX647^+^ CD27^hi^ CD38^hi^ CD4^−^ cells) were analyzed by flow cytometry. Spike-specific ASCs were intracellularly stained. All data analyses were performed with FlowJo 10.0 software (Tree Star, San Carlos, CA, USA).

### Statistical analysis

The Kolmogorov-Smirnov test was used to determine whether data had a normal distribution. Data are presented as the median and IQR (interquartile range) when variables had nonnormal distributions, and Mann–Whitney *U* tests were used to analyze two independent variables. For paired sample comparison, paired *t*-tests were used to analyze the differences between two groups. Differences among multiple groups were assessed by one-way analysis of variance, and Tukey’s test was used to assess differences between two groups at the same time point. Spearman’s rank correlation coefficient was used to measure the correlation between two different variables. All data analyses were performed using SPSS v.26 and GraphPad Prism v.8.0. All numerical data shown in this study were collected from at least three independent experiments.

### Supplementary information

SUPPLEMENTAL MATERIAL

## Data Availability

The data generated by the current study are available from the corresponding authors upon reasonable request.
