# Supplementary material for: SARS-CoV-2 spike-specific TFH cells exhibit unique responses in infected and vaccinated individuals
Source: Signal Transduct Target Ther. 2023 Oct 6;8:393. doi: 10.1038/s41392-023-01650-x (PMC10558553; doi:10.1038/s41392-023-01650-x)
Supplement: Supplementary file 1 — SUPPLEMENTAL MATERIAL [file 41392_2023_1650_MOESM1_ESM.docx]

Supplementary Materials for

**SARS-CoV-2 spike-specific T_FH_ cells exhibit unique responses in infected and vaccinated individuals**

Rongzhang He^1, 2 #^, Xingyu Zheng^1, 2 #^, Jian Zhang^2 #^, Bo Liu^2 #^, Qijie Wang^3 #^, Qian Wu^2, 4^, Ziyan Liu^2^, Fangfang Chang^4^, Yabin Hu^2^, Ting Xie^3^, Yongchen Liu^4^, Jun Chen^2^, Jing Yang^2^, Shishan Teng^2^, Rui Lu^2^, Dong Pan^2^, You Wang^2, 5^, Liting Peng^2^, Weijin Huang^6^, Velislava Terzieva^7^, Wenpei Liu^1, 2^, Youchun Wang^6 *^, Yi-Ping Li^4 *^, Xiaowang Qu^1, 2 *^

Correspondence to: [quxiaowang@163.com](mailto:quxiaowang@163.com); [lyiping@mail.sysu.edu.cn](mailto:lyiping@mail.sysu.edu.cn); [wangyc@nifdc.org.cn](mailto:wangyc@nifdc.org.cn)

**This PDF file includes:**

Supplementary Figure 1-6 with their legends

Supplementary Table 1- 5

Supplementary Figure

**Supplementary Fig 1. Gating strategy for spike-specific T_FH_ cell and subsets.** Gating strategy for spike-specific T_FH_ cells (CD154^+^ CXCR5^+^ CD4^+^ CD3^+^ T cells), spike-specific CXCR3^+^ T_FH_ cells (CD154^+^ CXCR3^+^ CXCR5^+^ CD4^+^ CD3^+^ T cells) and spike-specific CXCR3^-^ T_FH_ cells (CD154^+^ CXCR3^-^ CXCR5^+^ CD4^+^ CD3^+^ T cells).

**Supplementary Fig 2. Spike-specific T_FH_ cell and subset responses at 24 months in COVID-19 convalescents, as determined by CD137/CD154 assay.** Comparison of the frequencies of CD154^+^ CD137^+^ T_FH_ cells (n=5) (**a**), CD154^+^ CD137^+^ CXCR3^+^ T_FH_ cells (n=5) (**b**), and CD154^+^ CD137^+^ CXCR3^-^ T_FH_ cells (n=5) (**c**) from COVID-19 convalescents at 24 months upon BSA or spike protein stimulation. Paired *t*-test was used to compare the differences between the BSA and spike protein stimulation groups. P<0.05 was considered to be a two-tailed significant difference; ns, not significant.

**Supplementary Fig 3. Correlations among spike-specific antibody endpoint titers and neutralization titers in COVID-19 convalescents.** Correlations among spike-specific IgG (**a**), IgG1 (**b**), IgG3 (**c**), and IgA (**d**) endpoint titers and neutralization in COVID-19 convalescents at 2 months (n=25), 5 months (n=25), 8 months (n=25), 12 months (n=21), and 24 months (n=5). Spearman’s rank correlation coefficient was used to describe the association between the spike-specific endpoint titers and neutralization titers. P< 0.05 was considered to be a two-tailed significant difference.


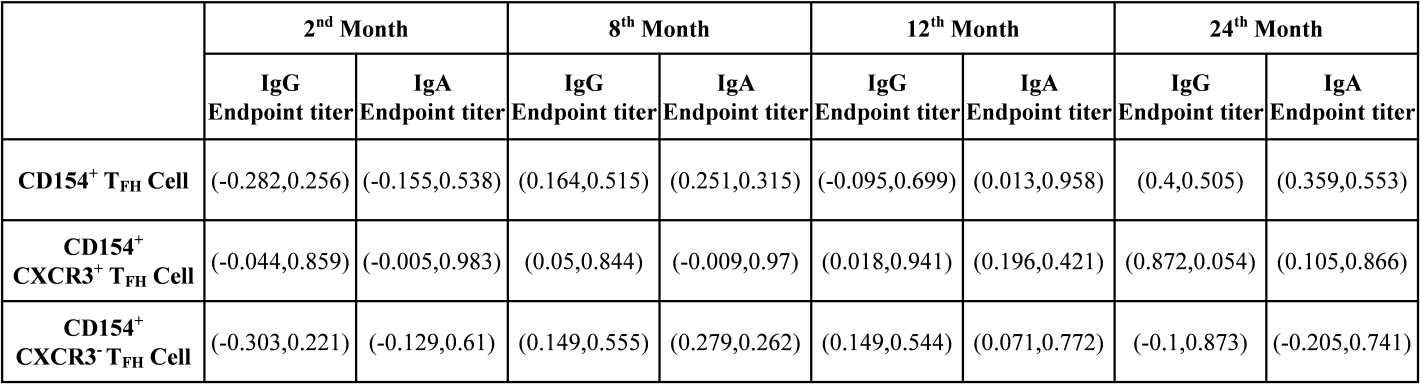


**Supplementary Fig 4. Correlations among spike-specific antibody endpoint titers and T_FH_ cell and subset responses in COVID-19 convalescents.** Correlations among spike-specific IgG and IgA endpoint titers and T_FH_ cell and subset responses (data presented with background subtracted) in COVID-19 convalescents at 2 months (n=18), 8 months (n=18), 12 months (n=19), and 24 months (n=5) after illness onset. Spearman’s rank correlation coefficient was used to describe the association between spike-specific endpoint titers and T_FH_ cell and subset responses. P< 0.05 was considered to be a two-tailed significant difference.

**Supplementary Fig 5. Correlation of spike-specific ASCs with spike-specific IgG in supernatants after coculture.** Correlation of spike-specific ASCs and the spike-specific IgG OD_450_ after coculturing T_FH_ cells with autologous memory B cells from convalescents (n=5) and vaccinated subjects (n=14) in the presence of SEB (100 ng/mL) for 6 days. Spearman’s rank correlation coefficient was used to describe the correlation between two different variables. P< 0.05 was considered to be a two-tailed significant difference*.*

**Supplementary Fig 6. *Ex vivo* CD154 expression by CXCR3^+^ and CXCR3^-^ T_FH_ cells in COVID-19 convalescents and inactivated vaccine recipients.** Comparison of *ex vivo* CD154^+^ CXCR3^+^ T_FH_ cell and CXCR3^-^ T_FH_ cell frequencies in COVID-19 convalescents (**a**) (n=72) and inactivated vaccine recipients (**b**) (n=105). Data are presented as mean ± SEM. Paired *t*-test was used to analyze the difference between the two groups. P< 0.05 was considered to be a two-tailed significant difference.

**Supplementary Tables**

**Supplementary Table 1. Baseline characteristics of participants with COVID-19 recruited in this study**

| **Patient** | A**ge**  **(years)** | **Sex** | **Disease severity** |  | | **2^nd^ month** | | **5^th^ month** | | **8^th^ month** | | | **12^th^ month** | | **24^th^ month** | | |
| --- | --- | --- | --- | --- | --- | --- | --- | --- | --- | --- | --- | --- | --- | --- | --- | --- | --- |
|  |  |  |  | **Vaccination** | | **Days*** | **PBMCs** | **Days*** | **PBMCs** | | **Days*** | **PBMCs** | **Days*** | **PBMCs** | | **Days*** | **PBMCs** |
|  |  |  |  |  | |  | **availability** |  | **availability** | |  | **availability** |  | **availability** | |  | **availability** |
| **Patient 1** | 29 | Male | Non severe | **-** | | 48 | **+** | 149 | **-** | 255 | | **+** | 382 | **+** | NA | | |
| **Patient 2** | 48 | Male | Non severe | **-** | | 42 | **+** | 143 | **-** | 249 | | **+** | 376 | **-** | NA | | |
| **Patient 3** | 45 | Female | Non severe | **-** | | 44 | **+** | 143 | **+** | 249 | | **-** | 387 | **+** | NA | | |
| **Patient 4** | 20 | Female | Non severe | **-** | | 54 | **+** | 149 | **+** | 255 | | **+** | NA | | NA | | |
| **Patient 5** | 50 | Female | Non severe | **-** | | 47 | **+** | 142 | **-** | 248 | | **-** | 382 | **-** | NA | | |
| **Patient 6** | 69 | Female | Non severe | **-** | | 52 | **-** | 179 | **+** | 248 | | **+** | 375 | **+** | NA | | |
| **Patient 7** | 46 | Female | Non severe | **-** | | 59 | **-** | 149 | **+** | 255 | | **+** | 375 | **+** | NA | | |
| **Patient 8** | 28 | Female | Non severe | **-** | | 54 | **+** | 144 | **+** | 250 | | **+** | NA | | NA | | |
| **Patient 9** | 21 | Female | Non severe | **-** | | 67 | **+** | 173 | **+** | 242 | | **+** | NA | | NA | | |
| **Patient 10** | 43 | Male | Non severe | **-** | | 48 | **+** | 186 | **+** | 255 | | **+** | 369 | **+** | NA | | |
| **Patient 11** | 23 | Male | Non severe | **-** | | 62 | **-** | 182 | **+** | 251 | | **-** | 382 | **+** | NA | | |
| **Patient 12** | 43 | Female | Non severe | **-** | | 63 | **+** | 190 | **+** | 259 | | **+** | 379 | **+** | NA | | |
| **Patient 13** | 30 | Female | Non severe | **-** | | 65 | **+** | 171 | **+** | 240 | | **+** | NA | | NA | | |
| **Patient 14** | 45 | Female | Non severe | **-** | | 76 | **-** | 182 | **+** | 251 | | **+** | 386 | **+** | NA | | |
| **Patient 15** | 22 | Male | Non severe | **-** | | 66 | **+** | 172 | **+** | 241 | | **+** | 367 | **+** | NA | | |
| **Patient 16** | 43 | Male | Non severe | **-** | | 57 | **+** | 184 | **+** | 253 | | **-** | 388 | **+** | 742 | | **+** |
| **Patient 17** | 47 | Male | Non severe | **-** | | 62 | **+** | 145 | **+** | 251 | | **-** | NA | | NA | | |
| **Patient 18** | 50 | Female | Non severe | **-** | | 58 | **+** | 153 | **+** | 259 | | **+** | 368 | **+** | NA | | |
| **Patient 19** | 47 | Female | Non severe | **-** | | 45 | **+** | 128 | **+** | 234 | | **+** | 371 | **+** | NA | | |
| **Patient 20** | 47 | Male | Severe | **-** | | 53 | **+** | 154 | **+** | 260 | | **+** | NA | | NA | | |
| **Patient 21** | 64 | Male | Severe | **-** | | 56 | **+** | 183 | **+** | 252 | | **+** | NA | | NA | | |
| **Patient 22** | 72 | Female | Severe | **-** | | 55 | **-** | 145 | **-** | 251 | | **+** | NA | | NA | | |
| **Patient 23** | 34 | Female | Severe | **-** | | 51 | **+** | 183 | **-** | 252 | | **+** | 378 | **+** | NA | | |
| **Patient 24** | 67 | Female | Severe | **-** | | 86 | **+** | 192 | **-** | 261 | | **+** | NA | | NA | | |
| **Patient 25** | 54 | Male | Severe | **-** | | 43 | **+** | 175 | **-** | 244 | | **+** | 361 | **+** | NA | | |
| **Patient 26** | 52 | Male | Non severe | **-** | | NA | | NA | | NA | | | 370 | **+** | NA | | |
| **Patient 27** | 22 | Female | Non severe | **-** | | NA | | NA | | NA | | | 370 | **+** | NA | | |
| **Patient 28** | 59 | Female | Severe | **-** | | NA | | NA | | NA | | | 381 | **+** | NA | | |
| **Patient 29** | 30 | Male | Non severe | **-** | | NA | | NA | | NA | | | 370 | **+** | NA | | |
| **Patient 30** | 64 | Female | Non severe | **-** | | NA | | NA | | NA | | | 381 | **+** | NA | | |
| **Patient 31** | 18 | Female | Non severe | **-** | | NA | | NA | | NA | | | 367 | **-** | NA | | |
| **Patient 32** | 43 | Male | Non severe | **-** | | NA | | NA | | NA | | | 381 | **-** | NA | | |
| **Patient 33** | 30 | Female | Non severe | **-** | | NA | | NA | | NA | | | 375 | **+** | NA | | |
| **Patient 34** | 43 | Male | Severe | **-** | | NA | | NA | | NA | | | NA | | 731 | | **+** |
| **Patient 35** | 30 | Female | Non severe | **-** | | NA | | NA | | NA | | | NA | | 706 | | **+** |
| **Patient 36** | 52 | Male | Non severe | **-** | | NA | | NA | | NA | | | NA | | 701 | | **+** |
| **Patient 37** | 43 | Female | Non severe | **-** | | NA | | NA | | NA | | | NA | | 701 | | **+** |
| Median  (IQR) | 43  (30.00-51.00) |  |  |  | 55.00  (48.00-62.50) | | | 171.00  (145.00-182.50) | | 251.00  (248.00-255.00) | | | 375.50  (370.00-381.75) | | 706.00  (701.00-736.50) | | |

***Specific day that samples were collected for each participant. In the manuscript, the 2^nd^, 5^th^, 8^th^, 12^th^ and 24^th^ month were used for simplicity. +: PBMCs were collected for this experiment; -: PBMCs were not collected; NA: Sample were not available. IQR: Interquartile range.**

**Supplementary Table 2. Baseline characteristics of vaccine recipients recruited in this study**

| **Vaccine recipient** | **Age (years)** | **Sex** |  | **PBMCs availability** | | | | | | | | |
| --- | --- | --- | --- | --- | --- | --- | --- | --- | --- | --- | --- | --- |
|  |  |  | **SARS-CoV-2 exposure and infection history** | **1^st^ dose**  **Day0** | **1^st^ dose**  **Day14** | **1^st^ dose**  **Day28** | **2^nd^ dose**  **Day14** | **2^nd^ dose**  **Day60** | **2^nd^ dose**  **Day150** | **Before Third dose** | | **After Third dose** |
| **Vaccine recipient 1** | 37 | Female | **-** | **-** | **+** | **-** | **-** | **+** | **-** | NA | NA | |
| **Vaccine recipient 2** | 33 | Female | **-** | **+** | **-** | **+** | **+** | **+** | **-** | NA | NA | |
| **Vaccine recipient 3** | 26 | Female | **-** | **+** | **-** | **+** | **+** | **+** | **+** | NA | NA | |
| **Vaccine recipient 4** | 26 | Female | **-** | **+** | **+** | **+** | **+** | **+** | **+** | NA | NA | |
| **Vaccine recipient 5** | 40 | Male | **-** | **+** | **+** | **+** | **+** | **+** | **+** | **+** | **+** | |
| **Vaccine recipient 6** | 30 | Female | **-** | **+** | **+** | **+** | **+** | **+** | **+** | NA | NA | |
| **Vaccine recipient 7** | 33 | Female | **-** | **+** | **+** | **+** | **-** | **+** | **+** | NA | NA | |
| **Vaccine recipient 8** | 35 | Female | **-** | **+** | **+** | **+** | **+** | **+** | **+** | NA | NA | |
| **Vaccine recipient 9** | 28 | Female | **-** | **+** | **+** | **+** | **+** | **+** | **+** | **+** | **+** | |
| **Vaccine recipient 10** | 25 | Female | **-** | **+** | **+** | **+** | **+** | **+** | **+** | **+** | **+** | |
| **Vaccine recipient 11** | 56 | Female | **-** | **+** | **+** | **+** | **+** | **+** | **+** | NA | NA | |
| **Vaccine recipient 12** | 42 | Male | **-** | **+** | **+** | **+** | **+** | **+** | **-** | **+** | **+** | |
| **Vaccine recipient 13** | 33 | Female | **-** | **+** | **+** | **+** | **+** | **+** | **-** | **+** | **+** | |
| **Vaccine recipient 14** | 31 | Male | **-** | **+** | **+** | **-** | **+** | **+** | **+** | **+** | **+** | |
| **Vaccine recipient 15** | 39 | Female | **-** | **+** | **+** | **+** | **+** | **+** | **+** | NA | NA | |
| **Vaccine recipient 16** | 36 | Female | **-** | **+** | **+** | **+** | **+** | **+** | **-** | **+** | **+** | |
| **Vaccine recipient 17** | 33 | Female | **-** | **+** | **+** | **+** | **+** | **+** | **-** | NA | NA | |
| **Vaccine recipient 18** | 51 | Male | **-** | **+** | **+** | **+** | **+** | **+** | **-** | **+** | **+** | |
| **Vaccine recipient 19** | 45 | Male | **-** | **+** | **+** | **+** | **+** | **+** | **+** | **+** | **+** | |
| **Vaccine recipient 20** | 32 | Female | **-** | **+** | **+** | **+** | **+** | **+** | **+** | **+** | **+** | |
| **Vaccine recipient 21** | 45 | Female | **-** | **+** | **+** | **+** | **+** | **+** | **+** | **+** | **+** | |
| **Vaccine recipient 22** | 34 | Female | **-** | **+** | **+** | **-** | **+** | **+** | **+** | NA | NA | |
| **Vaccine recipient 23** | 32 | Female | **-** | **+** | **+** | **+** | **+** | **+** | **+** | NA | NA | |
| **Vaccine recipient 24** | 36 | Female | **-** | **-** | **+** | **+** | **+** | **+** | **+** | **+** | **+** | |
| **Vaccine recipient 25** | 31 | Female | **-** | **+** | **+** | **+** | **+** | **+** | **+** | NA | NA | |
| **Vaccine recipient 26** | 40 | Female | **-** | **+** | **+** | **+** | **+** | **+** | **+** | **+** | **+** | |
| **Vaccine recipient 27** | 29 | Female | **-** | NA | NA | NA | NA | NA | NA | **+** | **+** | |
| **Vaccine recipient 28** | 48 | Male | **-** | NA | NA | NA | NA | NA | NA | **+** | **+** | |
| **Vaccine recipient 29** | 47 | Male | **-** | NA | NA | NA | NA | NA | NA | **+** | **+** | |
| **Vaccine recipient 30** | 49 | Male | **-** | NA | NA | NA | NA | NA | NA | **+** | **+** | |
| **Vaccine recipient 31** | 31 | Female | **-** | NA | NA | NA | NA | NA | NA | **+** | **+** | |
| **Vaccine recipient 32** | 49 | Female | **-** | NA | NA | NA | NA | NA | NA | **+** | **+** | |
| **Vaccine recipient 33** | 43 | Female | **-** | NA | NA | NA | NA | NA | NA | **+** | **+** | |
| **Vaccine recipient 34** | 38 | Female | **-** | NA | NA | NA | NA | NA | NA | **+** | **+** | |
| **Vaccine recipient 35** | 38 | Female | **-** | NA | NA | NA | NA | NA | NA | **+** | **+** | |
| **Vaccine recipient 36** | 37 | Female | **-** | NA | NA | NA | NA | NA | NA | **+** | **+** | |
| **Vaccine recipient 37** | 28 | Female | **-** | NA | NA | NA | NA | NA | NA | **+** | **+** | |
| **Median**  **(IQR)** | 36  (31-42.5) |  |  |  |  |  |  |  |  |  |  | |

**+: PBMCs were collected for this experiment; -: PBMCs were not collected; NA: Sample were not available. IQR: Interquartile range.**

**Supplementary Table 3. Sample information used in Figure 5**

| **Vaccine recipient** | **Age (years)** | **Sex** | **SARS-CoV-2 exposure and infection history** | **Vaccination** |
| --- | --- | --- | --- | --- |
| **Vaccine recipient 38** | 26 | Male | - | Two-dose |
| **Vaccine recipient 39** | 45 | Female | - | Two-dose |
| **Vaccine recipient 40** | 35 | Female | - | Two-dose |
| **Vaccine recipient 41** | 52 | Female | - | Two-dose |
| **Vaccine recipient 42** | 37 | Female | - | Two-dose |
| **Vaccine recipient 43** | 35 | Male | - | Two-dose |
| **Vaccine recipient 44** | 33 | Female | - | Two-dose |
| **Vaccine recipient 45** | 33 | Female | - | Two-dose |
| **Vaccine recipient 46** | 37 | Female | - | Two-dose |
| **Vaccine recipient 47** | 28 | Female | - | Third dose |
| **Vaccine recipient 48** | 53 | Female | - | Third dose |
| **Vaccine recipient 49** | 42 | Male | - | Third dose |
| **Vaccine recipient 50** | 37 | Female | - | Third dose |
| **Vaccine recipient 51** | 50 | Female | - | Third dose |
| **Vaccine recipient 52** | 47 | Male | - | Third dose |
| **Vaccine recipient 53** | 37 | Female | - | Third dose |
| **Vaccine recipient 54** | 36 | Female | - | Third dose |
| Median  (IQR) | 37  (34-46) |  |  |  |

**-: Sample was not infected with COVID-19; IQR: Interquartile range.**

**Supplementary Table 4. Sample information used in Figure 6**

| **Patient** | | | | **Vaccine recipient** | | | | | |
| --- | --- | --- | --- | --- | --- | --- | --- | --- | --- |
| **Patient** | **Age (years)** | **Sex** | **Vaccination** | **Vaccine recipient** | | **Age (years)** | **Sex** | **SARS-CoV-2 exposure and infection history** | **Vaccination** |
| **Patient 38** | 72 | Female | - | **Vaccine recipient 55** | | 40 | Male | - | Two-dose |
| **Patient 39** | 34 | Female | - | **Vaccine recipient 56** | | 35 | Female | - | Two-dose |
| **Patient 40** | 29 | Male | - | **Vaccine recipient 57** | | 28 | Female | - | Two-dose |
| **Patient 41** | 48 | Male | - | **Vaccine recipient 58** | | 33 | Female | - | Two-dose |
| **Patient 42** | 29 | Male | - | **Vaccine recipient 59** | | 33 | Female | - | Two-dose |
| **Patient 43** | 29 | Female | - | **Vaccine recipient 60** | | 32 | Female | - | Two-dose |
| **Patient 44** | 43 | Male | - | **Vaccine recipient 61** | | 31 | Female | - | Two-dose |
| **Patient 45** | 72 | Female | - | **Vaccine recipient 62** | | 31 | Female | - | Two-dose |
| **Patient 46** | 46 | Female | - | **Vaccine recipient 63** | | 34 | Female | - | Two-dose |
| **Patient 47** | 34 | Female | - | **Vaccine recipient 64** | | 33 | Female | - | Two-dose |
| **Patient 48** | 43 | Male | - | **Vaccine recipient 65** | | 28 | Female | - | Two-dose |
| **Patient 49** | 51 | Female | - | **Vaccine recipient 66** | | 29 | Female | - | Two-dose |
| **Patient 50** | 21 | Female | - | **Vaccine recipient 67** | | 29 | Female | - | Two-dose |
| **Patient 51** | 30 | Female | - | **Vaccine recipient 68** | | 34 | Male | - | Two-dose |
| **Patient 52** | 45 | Female | - | **Vaccine recipient 69** | | 29 | Female | - | Two-dose |
| **Patient 53** | 43 | Female | - | **Vaccine recipient 70** | | 45 | Female | - | Third dose |
| **Patient 54** | 50 | Female | - | **Vaccine recipient 71** | | 25 | Female | - | Third dose |
| **Patient 55** | 72 | Female | - | **Vaccine recipient 72** | | 36 | Female | - | Third dose |
| **Patient 56** | 47 | Male | - | **Vaccine recipient 73** | | 40 | Female | - | Third dose |
| **Patient 57** | 28 | Female | - | **Vaccine recipient 74** | | 42 | Male | - | Third dose |
| **Patient 58** | 65 | Female | - | **Vaccine recipient 75** | | 33 | Female | - | Third dose |
| **Patient 59** | 53 | Female | - | **Vaccine recipient 76** | | 51 | Male | - | Third dose |
| **Patient 60** | 30 | Male | - | **Vaccine recipient 77** | | 29 | Female | - | Third dose |
| **Patient 61** | 44 | Male | - | **Vaccine recipient 78** | | 28 | Female | - | Third dose |
| **Patient 62** | 32 | Female | - | **Vaccine recipient 79** | | 47 | Male | - | Third dose |
| **Patient 63** | 21 | Female | - | **Vaccine recipient 80** | | 31 | Male | - | Third dose |
| **Patient 64** | 50 | Female | - | **Vaccine recipient 81** | | 32 | Male | - | Third dose |
|  |  |  |  | **Vaccine recipient 82** | | 51 | Female | - | Third dose |
|  |  |  |  | **Vaccine recipient 83** | | 24 | Female | - | Third dose |
| Median  (IQR) | 43  (30-50) |  |  | Median  (IQR) | 33  (29-38) | |  |  |  |
|  |  |  |  |  |  | |  |  |  |

**-: Patient was no vaccination or Vaccine recipient was not infected with COVID-19; IQR: Interquartile range.**

**Supplementary Table 5. Sample information used in Figure 7**

| **Patient** | | | | **Vaccine recipient** | | | | | |
| --- | --- | --- | --- | --- | --- | --- | --- | --- | --- |
| **Patient** | **Age (years)** | **Sex** | **Vaccination** | **Vaccine recipient** | **Age (years)** | **Sex** | **SARS-CoV-2 exposure and infection history** | **Vaccination** | |
|  |  |  |  |  |  |  |  | **Two-dose** | **Third dose** |
| **Patient 65** | 82 | Male | - | **Vaccine recipient 84** | 38 | Male | - | + | + |
| **Patient 66** | 29 | Male | - | **Vaccine recipient 85** | 49 | Male | - | + | + |
| **Patient 67** | 48 | Male | - | **Vaccine recipient 86** | 33 | Female | - | + | + |
| **Patient 68** | 75 | Male | - | **Vaccine recipient 87** | 45 | Female | - | + | + |
| **Patient 69** | 50 | Female | - | **Vaccine recipient 88** | 25 | Female | - | + | + |
| **Patient 70** | 41 | Female | - | **Vaccine recipient 89** | 33 | Male | - | + | + |
| **Patient 71** | 49 | Female | - | **Vaccine recipient 90** | 29 | Female | - | + | + |
| **Patient 72** | 24 | Female | - | **Vaccine recipient 91** | 38 | Female | - | + | - |
| **Patient 73** | 54 | Female | - | **Vaccine recipient 92** | 48 | Male | - | + | - |
| **Patient 74** | 46 | Female | - | **Vaccine recipient 93** | 46 | Male | - | + | - |
| **Patient 75** | 42 | Female | - | **Vaccine recipient 94** | 33 | Female | - | + | - |
| **Patient 76** | 29 | Male | - | **Vaccine recipient 95** | 28 | Female | - | + | - |
| **Patient 77** | 53 | Female | - |  |  |  |  |  |  |
| **Patient 78** | 11 | Female | - |  |  |  |  |  |  |
| Median | 47 |  |  | Median | 35.5 |  |  |  |  |
| (IQR) | (29-53.25) |  |  | (IQR) | (30-45.75) |  |  |  |  |

**-: Patient was no vaccination or Vaccine recipient was not infected with COVID-19; IQR: Interquartile range.**
